# NR2F6 regulates stem cell hematopoiesis and myelopoiesis in mice

**DOI:** 10.3389/fimmu.2024.1404805

**Published:** 2025-01-07

**Authors:** Johannes Woelk, Hamsa Narasimhan, Christa Pfeifhofer-Obermair, Barbara U. Schraml, Natascha Hermann-Kleiter

**Affiliations:** ^1^ Institute of Cell Genetics, Department for Genetics and Pharmacology, Medical University of Innsbruck, Innsbruck, Austria; ^2^ Institute for Immunology, Faculty of Medicine, Ludwig-Maximilians-Universität (LMU) Munich, Munich, Germany; ^3^ Institute of Cardiovascular Physiology and Pathophysiology at the Walter-Brendel-Centre of Experimental Medicine, Faculty of Medicine, Ludwig-Maximilians-Universität (LMU) Munich, Munich, Germany; ^4^ Department of Internal Medicine II (Infectious Diseases, Immunology, Rheumatology, Pneumology), Medical University of Innsbruck, Innsbruck, Austria

**Keywords:** nuclear receptor NR2F6, bone marrow, hematopoiesis, HSC, GMP, MDP, pre-cDC2, myeloid compartment

## Abstract

Nuclear receptors regulate hematopoietic stem cells (HSCs) and peripheral immune cells in mice and humans. The nuclear orphan receptor NR2F6 (EAR-2) has been shown to control murine hematopoiesis. Still, detailed analysis of the distinct stem cell, myeloid, and lymphoid progenitors in the bone marrow in a genetic loss of function model remains pending. In this study, we found that adult germline *Nr2f6*-deficient mice contained increased percentages of total long-term and short-term HSCs, as well as a subpopulation within the lineage-biased multipotent progenitor (MPP3) cells. The loss of NR2F6 thus led to an increase in the percentage of LSK^+^ cells. Following the differentiation from the common myeloid progenitors (CMP), the granulocyte-monocyte progenitors (GMP) were decreased, while monocyte-dendritic progenitors (MDP) were increased in *Nr2f6*-deficient bone marrow. Within the pre-conventional dendritic progenitors (pre-cDCs), the subpopulation of pre-cDC2s was reduced in the bone marrow of *Nr2f6*-deficient mice. We did not observe differences in the development of common lymphoid progenitor populations. Our findings contrast previous studies but underscore the role of NR2F6 in regulating gene expression levels during mouse bone marrow hematopoiesis and myelopoiesis.

## Introduction

Hematopoietic stem cells (HSCs) possess the remarkable capability to undergo self-renewal or differentiate and give rise to the diverse array of blood and immune cells throughout adult life (1–4). Within mammals, HSCs reside quiescently in the bone marrow, strategically positioned in specialized microenvironments or niches that facilitate their maintenance and regulate production (4). Functionally, HSCs are characterized by their capacity for serial engraftment in transplanted recipients, enabling the regeneration of the entire blood system, an intrinsic measure of their self-renewal activity (5). Given the post-mitotic nature of most mature hematopoietic cells and their brief half-life, the continual generation of mature blood cells from precursor cells is mandatory (6). Disruptions in HSC function can result in bone marrow failure or contribute to the progression of pre-malignant states and blood cancers (7).

The hematopoietic system is capable of rapid adaptation to hematopoietic stress, such as bleeding, severe infection, or tumor growth, by increasing cellular output several-fold above steady-state levels to meet the higher demand for the respective blood cell type, a phenomenon known as emergency hematopoiesis (8–11). Furthermore, bone marrow (BM)-mediated trained innate immunity has been recently characterized as a state of heightened immune responsiveness of hematopoietic stem and progenitor cells (HSPC) and their myeloid progeny, which can result in inflammatory comorbidities (12). Homeostatic and emergency myelopoiesis, by which these cells arise, primarily relies on myelopoietic cytokines like M-CSF, GM-CSF, G-CSF, and interferons (13–15).

Hematopoiesis begins in the yolk sac, undergoes a transient phase in the fetal liver, and culminates in definitive hematopoiesis within the bone marrow and thymus (16–18). The interplay between fetal development and bone marrow-derived adult hematopoiesis significantly influences the organ-specific composition of immune cells in steady-state conditions (19, 20). While tissue-resident macrophages self-renew locally, independent of adult hematopoiesis, short-lived monocyte-derived macrophages originate from adult hematopoietic stem cells and primarily accumulate in inflamed lesions (19–22). Conventional dendritic cells (cDCs), critical for innate and adaptive immune responses, comprise two functionally distinct lineages, namely cDC1 and cDC2 (23–25).

Plasmacytoid DCs (pDCs) represent a distinct family of innate type I IFN-producing lymphoid cells, different from the cDC lineage, recently defined to arise from both the myeloid and the lymphoid lineage (26–28). Common lymphoid progenitors (CLP) give rise to T cells, innate lymphoid cells (ILC) containing natural killer (NK) cells, B cells, and pDCs (27–30).

The nuclear receptor (NR) superfamily, consisting of 48 primarily ligand-activated transcription factors, plays a pivotal role in diverse physiological events, including stem cell differentiation and immune cell regulation (31–34). Numerous NR family members such as the retinoic acid receptor (RAR), RAR/retinoid X receptor (RXR) heterodimers, the peroxisome proliferator-activated receptors (PPAR), or nuclear receptors NR4A3 and NR4A1 are recognized regulators of HSCs fate in both mice and humans (3), [31-39]. In parallel, the nuclear receptor corepressor 1 (NCoR1) regulates hematopoiesis and leukemogenesis *in vivo* (40).

In the context of bone marrow hematopoiesis, the role of the orphan nuclear receptor NR2F6 (EAR-2) has been investigated both *in vitro* and *in vivo* using overexpression and silencing approaches, as well as chimeric models (41) (42). Ichim et al., demonstrated that *Nr2f6* (EAR-2) expression is higher in long-term hematopoietic stem cells compared to short-term hematopoietic stem and progenitor cells (41). A retroviral transfection model revealed that NR2F6 (EAR-2) inhibits hematopoietic cell differentiation *in vitro* and *in vivo* and induces myeloid dysplasia in mice (41). In *ex vivo* bone marrow culture, NR2F6 (EAR-2) over-expression reduced the number of colony forming cells, while silencing of NR2F6 (EAR-2) increased colony size, suggesting that NR2F6 (EAR-2) limits hematopoietic differentiation (41). *In vitro* suspension culture confirmed that NR2F6 (EAR-2) inhibits granulocytic and monocytic differentiation, reducing the number of BFU-E and CFU-GM colonies and the size of erythroid and myeloid colonies, whereas knockdown of NR2F6-induced granulocytic differentiation (41, 42). These studies suggest that down-regulation of NR2F6 (EAR-2) is essential for hematopoietic differentiation and that unregulated expression of NR2F6 (EAR-2) blocks this process (42).

However, our analysis of germline *Nr2f6*-deficient mice shows unaltered B cell development in the bone marrow and thymic T cell development (43). Previous research established NR2F6 as an intracellular immune checkpoint in peripheral T cells during experimental autoimmunity, germinal center responses, bacterial infection, and cancer immune surveillance (43–49). Our previous studies found that *Nr2f6*-deficient mice develop spontaneous colitis by one year of age but do not show signs of inflammation during the first 3 months (50). Nonetheless, the impact on lymphoid and myeloid progenitor populations in the bone marrow as well as the myeloid compartment in healthy *Nr2f6*-deficient mice, remains undefined.

Therefore, we aimed to investigate the roles of NR2F6 in distinct stem cell, myeloid, and lymphoid progenitor populations in the bone marrow of healthy germline *Nr2f6*-deficient mice, independent of chimera experiments or transduction approaches, being aware of the limitations of using a germline-deficient mouse model. Given the ongoing high-throughput screening for compounds targeting NR2F6, a deeper understanding of its functional roles in hematopoiesis is crucial (51).

## Materials and methods

### Mice

*Nr2f6-*deficient mice on the C57BL/6N background were bred in-house under specific pathogen-free conditions (44). The animals were maintained at room temperature with water and food provided *ad libitum*. The experiments were performed with 8-12 week-old mice, for each individual experiment, mice were age and sex-matched in a non-randomized manner, with a minimum of 3 mice per group. The ethics committee of the Medical University of Innsbruck and the Austrian Federal Ministry of Education Science and Research approved and authorized all animal procedures (GZ: 2021-0.406.862; GZ: 2021-0.406.863).

### Organ Harvest

Adult mice were terminally anesthetized by intraperitoneal (i.p.) injection of 200 µL Ketasol (100 mg/mL, Livisto)/Xylasol (20 mg/mL, Livisto) solution. Bone marrow was harvested from the tibia and femur. Briefly, bones were isolated under sterile conditions; one end of the bone was cut open and placed into a 0.5 ml tube containing a hole in the bottom (18-21 G needle puncture), which was put into a 1.5 ml tube. BM was collected by centrifugation of the isolating device in a table-top centrifuge at 5,000 × g for 20 s (52). The supernatant was sucked off or collected for cytokine measurements. Spleens were homogenized through a 100 μm cell strainer in FACS Buffer (PBS (Sigma Aldrich-Aldrich, P5493-1L) supplemented with 3% FBS, 1% Penicillin/Streptomycin (Sigma-Aldrich, A2213)). Blood was collected via the femoral artery after sacrifice. Red blood cells were lysed in erythrocyte lysis buffer, as described previously (49). Subsequently, single-cell suspension was filtered through a 70µm cell strainer prior to further processing.

### Flow cytometry

If the FC receptors CD16 and CD32 were not specifically targeted, the cells were incubated for 15 min with FcR block (anti-CD16/32; BD Biosciences) in FACS buffer. The cells were then incubated with fluorochrome-conjugated or biotinylated monoclonal antibodies for 30 min at 4°C in FACS buffer. After washing with PBS, we used the Fixable Viability Stain 780 (BD Biosciences, #565388) or Fixable Viability Stain 575V (BD Bioscience, #565694) for 10 min at 4°C to stain dead cells. If necessary, cells were incubated for 30 minutes with streptavidin-conjugated antibodies. The cells were then washed and resuspended in FACS buffer for subsequent flow cytometry analysis. Flow-cytometric analysis was conducted with BD LSRFortessa^™^ (BD Biosciences, Germany) or BD FACSCanto II BD Biosciences, Germany), followed by data analysis with the FlowJo software (BD Biosciences, New Jersey, v10.8.1). The flow cytometric data underwent quality control procedures, and any irregularities were removed. Only data of sufficient quality were utilized in subsequent analyses. See **Supplementary Table 1** for details of the markers used to identify stem and progenitor populations in the bone marrow and **Supplementary Table 2** for the full list of antibodies used for flow cytometry.

### Vet ABC ™Hematology Analyzer

12µl of blood was used to investigate blood parameters using a Vet ABC ™Hematology Analyzer.

### CFU assay

According to the manufacturer’s protocol, CFU assays were conducted using the mouse methylcellulose complete media (R&D, HSC007). In brief, 2.5x10^4^ bone marrow cells in 1.1 ml of mouse methylcellulose complete media were seeded in 35 mm cell culture dishes (triplicates). Eight days post-seeding, dishes containing CFUs were screened (Olympus CKX53), and movies of the complete dish were made (Jenoptik Optical Systems GmbH, Gryphax, Version 2.2.0.1234). Movies were blinded and randomized and CFUs on the plates were counted manually.

### Cytokine measurements

GM-CSF, M-CSF, G-CSF, and IL-1β cytokine levels from mouse bone marrow supernatant (see organ harvest) were analyzed with LEGENDplex™ Kit (mix and match) according to the instruction manual.

### Statistical analysis

Statistical analysis was performed using Prism 8.0 and 10.2.3. Unless otherwise indicated, experiments were repeated at least two times using a minimum of 3 mice per group. The Shapiro–Wilk test evaluated the normality of our data. When normally distributed, we performed statistical analysis with an unpaired Student’s *t*-test for samples with equal variance (*F* test) or an unpaired *t*-test with Welch’s correction for datasets with different variances. A Mann–Whitney U test was used if data were not normally distributed. Triplicates in the CFU assay were tested using a nested t-test. Results are shown as median ± IQR with whiskers from min. to max. Outliers were excluded using ROUT (Q=1%) in GraphPad Prism. A *p*-value < 0.05 was considered statistically significant. *p < 0.05; **p < 0.01, ***p < 0.001.

## Results

### HSC development is altered in *Nr2f6*-deficient bone marrow

In mice, hematopoietic stem cells (HSCs) in the bone marrow are found in the live Lin^−^Sca-1^+^c-Kit^+^ (LSK^+^) fraction, which includes both the hematopoietic stem and progenitor cell (HSPC) and lymphoid-myeloid primed progenitor (LMPP) populations (5). The HSPC population can be further divided into long-term (LT) LT-HSCs (LSK^+^CD135^−^CD48^−^CD150^+^) and short-term (ST) ST-HSCs (LSK^+^CD135^−^CD48^−^CD150^-^) (5, 53, 54). HSCs produce distinct parallel subsets of lineage-biased multipotent progenitors (MPPs), which coordinate myeloid and lymphoid lineage outputs in response to hematopoietic demands (5). MPP2s (LSK^+^CD135^−^CD48^+^CD150^+^) and MPP3s (LSK^+^CD135^−^CD48^+^CD150^-^) are contained within HSPCs (LSK^+^CD135^−^), in contrast, the MPP4 (CD48^+^CD150^-^) population is contained within the LMPP (LSK^+^CD135^+^) population (5).

**Figure 1:**
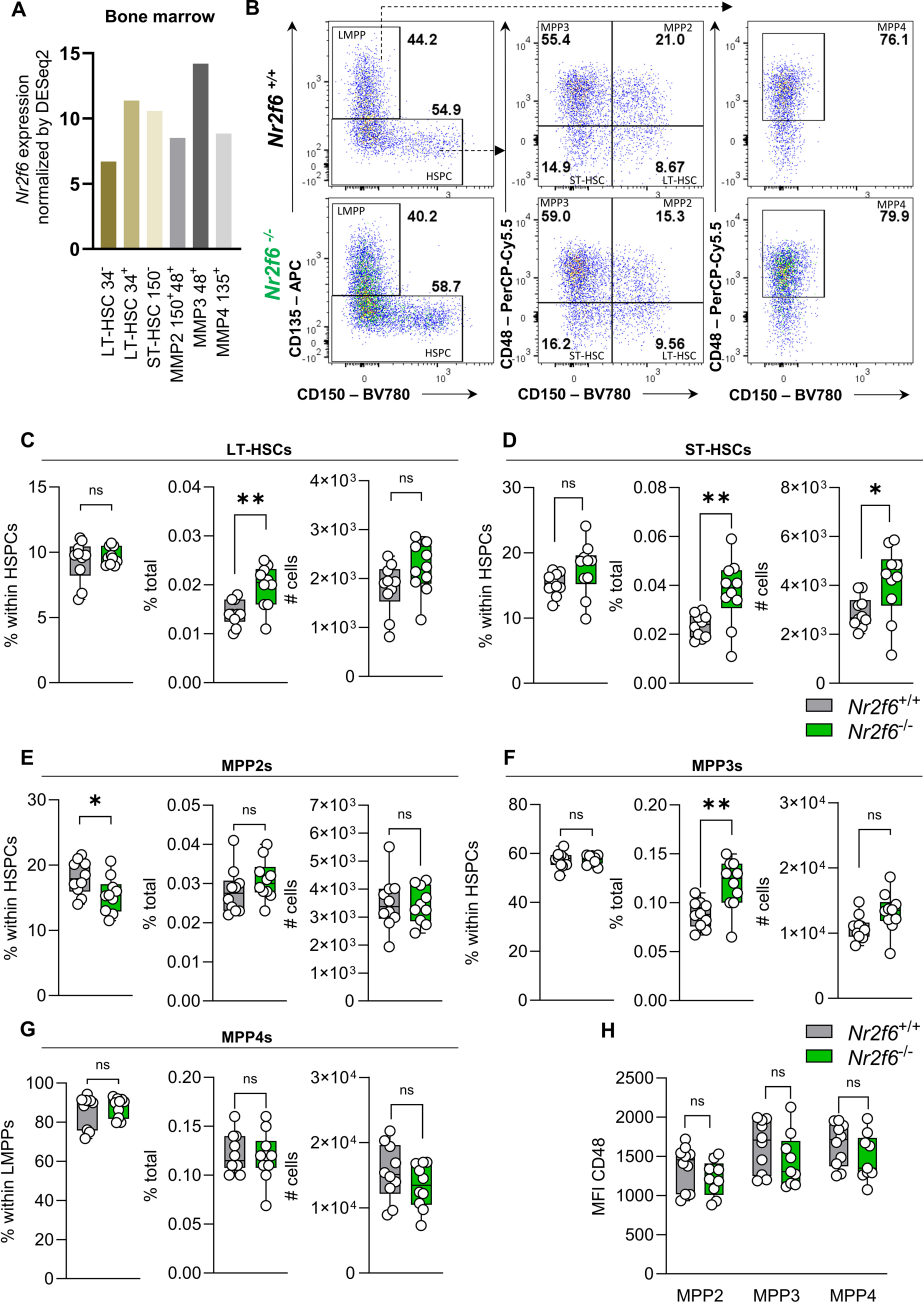
HSC and MPP development is altered in *Nr2f6*-deficient bone marrow. **(A)** Relative *Nr2f6* expression normalized by DESeq2 in different bone marrow progenitor populations such as CD34 negative long-term (LT-HSCs CD34^-^), CD34 positive long-term (LT-HSCs CD34^+^), and short-term (ST-HSCs CD150^-^) hematopoietic stem cells, as well as multipotent progenitor (MPP2 CD48^+^CD150^+^; MPP3 CD48^+^ and MPP4 CD135^+^) populations as defined by the Immgen consortium (55). **(B)** Representative dot plots of bone marrow-derived live (Lin)^−^CD127^-^LSK^+^CD135^+^CD150^-^ (LMPP), CD135^-^ (HSPC), CD48^-^CD150^+^ (LT-HSC), CD48^-^CD150^-^ (ST-HSC), CD48^+^CD150^+^ (MPP2), CD48^+^CD150^-^ (MPP3) and LMPP derived CD48^+^CD150^-^ (MPP4) populations from wild-type (*Nr2f6* ^+/+^) or *Nr2f6*-deficient (*Nr2f6* ^-/-^) mice. **(C-G)** Quantification of the percent of parent, percent of total and total cell numbers of live (Lin)^−^CD127^-^ HSPC derived CD48^-^CD150^+^ (LT-HSC) **(C)**, CD48^-^CD150^-^ (ST-HSC) **(D)**, CD48^+^CD150^+^ (MPP2) **(E)**, CD48^+^CD150^-^ (MPP3) **(F)** and LMPP derived CD48^+^CD150^-^ (MPP4) **(G)** populations from wild-type (*Nr2f6* ^+/+^) or *Nr2f6*-deficient (*Nr2f6* ^-/-^) mice. **(H)** Quantification of the CD48 MFI in the MPP2, MPP3 and MPP4 population from wild-type (*Nr2f6* ^+/+^) or *Nr2f6*-deficient (*Nr2f6* ^-/-^) mice. Representative data are shown as pooled experiments of at least three independent experiments, total *n* = 10/9-10 (*Nr2f6^+/+^*)/(*Nr2f6^-/-^*). Each dot represents the data of an individual mouse. Results are shown as mean **(A)**, or median ± IQR, with whiskers from min. to max. Outliers were excluded using ROUT (Q=1%) in GraphPad Prism. The Shapiro-Wilk test evaluated the normality of data. Asterisks indicate statistically significant differences between genotypes calculated using the Student’s *t*-test or Mann-Whitney *U* test for non-parametric data. A *p*-value < 0.05 was considered statistically significant, *0.05, **0.01 (See also **Supplementary Figure S1**).

Analysis of Immgen database data revealed *Nr2f6* expression in various bone marrow stem cell populations (**Figure 1A**) (55). To explore the role of NR2F6 in adult mouse hematopoiesis, we isolated the bone marrow from the femurs and the tibias of age and sex-matched wild-type or germline *Nr2f6*-deficient mice and characterized progenitor populations utilizing flow cytometry (**Supplementary Figure 1A**). We determined the percentage of parent gate, the percentage of total (out of live, single cells), and the live cell numbers of each individual population within the bone marrow of both genotypes. The size and weight of the femur and tibia and the total bone marrow counts were not altered between the genotypes (**Supplementary Figure 1B**).

While the percentages of parent long-term (LT) and short-term (ST) hematopoietic stem cell (HSC) populations were unchanged, the percentages of total LT-HSCs and ST-HSCs were significantly higher in the bone marrow of *Nr2f6*-deficient mice when compared to wild-type controls (**Figures 1B–D**, **Supplementary Figure 1C**). Along this line, total cell numbers of ST-HSC were significantly increased, with a trend to an enhanced LT-HSC population in *Nr2f6*-deficient mice (**Figures 1C, D**).

Within the distinct parallel subsets of lineage-biased multipotent progenitors (MPP) derived from the HSC compartment, we did not observe differences in percentages of total and MPP2 cell numbers in the bone marrow of *Nr2f6*-deficient mice. However, the percentage of parent MPP2 population was reduced in *Nr2f6*-deficient mice compared to wild-type controls (**Figure 1E**). In contrast, we did not observe an alteration in the percentage of parent MPP3s, although the percentage of total MPP3s significantly increased compared to the wild-type (**Figure 1F**). Consequently, although not statistically significant, we also observed a trend to enhance total MPP3 cell numbers in the bone marrow of *Nr2f6*-deficient mice (**Figure 1F**). No differences between the genotypes were observed within the MPP4 population, defined in the fms-like tyrosine kinase 3 (FLT-3; CD135) positive LMPP compartment (**Figure 1G**). Along this line, MFI levels of CD48 and CD150 in all populations were similar between the genotypes (**Figure 1H**, **Supplementary Figure 1D**). To assess the proliferation capacity of these single stem cell populations, we performed Ki-67 staining. Our results demonstrated a significant reduction in proliferation in the *Nr2f6*-deficient LT and ST-HSC populations (**Supplementary Figures 1E, F**). A comparable trend, although not reaching statistical significance, was observed in MPP2, MPP3, and MPP4 populations (**Supplementary Figure 1F**). In parallel to Ichim et al. (42), we investigated Lin^+^CD11b^+^ cells within the BM of *Nr2f6*-deficient mice and observed an increase in the percentage of total but not cell numbers of the Lin^+^CD11b^+^ population (**Supplementary Figure 1G**).

### Loss of NR2F6 induces changes in LSK^+^ and Sca-1^+^ cell proportions

The alterations observed in the MPP populations of *Nr2f6*-deficient mice prompted us to ask if the output among hematopoietic lineages was altered by loss of NR2F6. Therefore, we investigated potential changes in hematopoietic stem and progenitor cells (HSPC), the lymphoid-primed multipotent progenitor (LMPP), and Lin^+^Sca-1^+^c-Kit^+^ (LSK) populations in the bone marrow.

**Figure 2:**
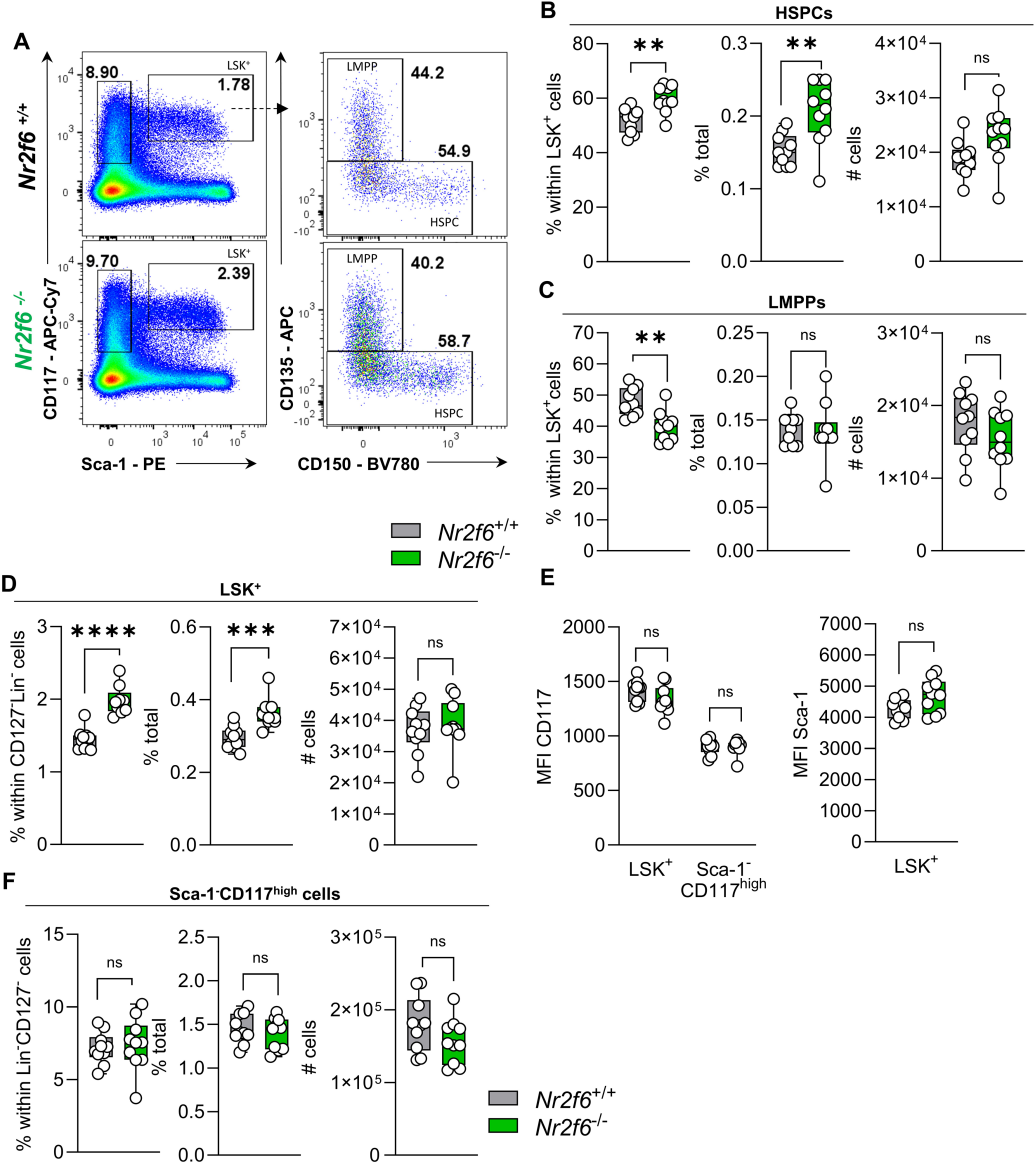
Loss of NR2F6 induces changes in LSK^+^ and Sca-1^+^ cell proportions. **(A)** Representative dot plots of bone marrow-derived live (Lin)^−^CD127^-^Sca-1^-^c-kit (CD117)^high^ and Sca-1^+^c-kit (CD117)^+^ (LSK^+^) positive cell populations together with LSK^+^ CD135^+^CD150^-^ (LMPP) and CD135^-^ (HSPC) populations from wild-type (*Nr2f6* ^+/+^) or *Nr2f6*-deficient (*Nr2f6* ^-/-^) mice. **(B-D)** Quantification of the percent of parent, percent of total and total cell numbers of CD135^-^ (HSPC) **(B)** and CD135^+^CD150^-^ (LMPP) **(C)** derived from live (Lin)^−^CD127^-^Sca-1^+^c-kit (CD117)^+^ LSK^+^ **(D)** populations from wild-type (*Nr2f6* ^+/+^) or *Nr2f6*-deficient (*Nr2f6* ^-/-^) mice. **(E)** Quantification of the MFI of c-kit (CD117) within LSK^+^ and Sca-1^-^c-kit (CD117)^high^ populations and MFI of Sca-1 in LSK^+^ cells from wild-type (*Nr2f6* ^+/+^) or *Nr2f6*-deficient (*Nr2f6* ^-/-^) mice. **(F)** Quantification of the percent of parent, percent of total and total cell numbers of live (Lin)^−^CD127^-^ Sca-1^-^c-kit (CD117)^high^ populations from wild-type (*Nr2f6* ^+/+^) or *Nr2f6*-deficient (*Nr2f6* ^-/-^) mice. Representative data are shown as pooled experiments of at least three independent experiments, total *n* = 10/9-10 (*Nr2f6^+/+^*)/(*Nr2f6^-/-^*). Each dot represents the data of an individual mouse. Results are shown median ± IQR with whiskers from min. to max. Outliers were excluded using ROUT (Q=1%) in GraphPad Prism. The Shapiro-Wilk test evaluated the normality of data. Asterisks indicate statistically significant differences between genotypes calculated using the Student’s *t*-test or Mann-Whitney *U* test for non-parametric data. A *p*-value < 0.05 was considered statistically significant, *0.05, **0.01, ***0.001, ****0.0001 (See also **Supplementary Figure S2**).

Notably, the percentages of parent and total HSPCs were significantly enhanced in *Nr2f6*-deficient mice, resulting in a trend to increased total HSPC cell numbers, albeit not statistically significant, compared to wild-type controls (**Figures 2A, B**).

In contrast, the percentage of the parent LMPP population was significantly reduced in *Nr2f6*-deficient mice, but the percentage of total and cell numbers were similar to wild-type, independent of CD135 expression (MFI) levels (**Figures 2A, C**, **Supplementary Figure 2A**).

The shift in percentages of total HSPCs populations, namely LT-HSCs, ST-HSCs and MPP3 cells, but not MPP2 cells, results in increased percentages of parent and total LSK^+^ cells in the bone marrow of *Nr2f6*-deficient mice (**Figures 2A, D**). The expression levels (MFI) of CD117 and Sca-1 within the LSK^+^ populations were comparable between the genotypes (**Figure 2E**).

We did not observe differences in the Lin^-^CD127^-^ pre-gate (**Supplementary Figure 2B**). Additionally, the percentage of parent and total as well as total cell numbers of the Sca-1^-^c-Kit^+^ (CD117^+^) population were similar between the genotypes (**Figures 2A, F**). Ki-67 staining revealed no significant differences in Ki-67 levels within HSCPC LMPP and LSK+ cells (**Supplementary Figure 2C**). Taken together, the absence of NR2F6 leads to an increase in the percentage of total LT and ST-HSCs and MMP3 cells, resulting in an increased percentage of total HSPCs and LSK^+^ cells (**Supplementary Figure 2D**).

### GMP but not CMP or CLP populations are reduced in *Nr2f6*-deficient bone marrow

Whereas the common lymphoid progenitor (CLP) population (CD135^+^CD127^+^) is derived from Lin^−^Sca-1^+^c-Kit^lo/int^ cell population, common myeloid progenitors (CMPs) are contained in the Lin^-^CD127^-^Sca-1^-^c-Kit^+^CD115^-^ cell fraction and give rise to granulocyte-monocyte (macrophage) progenitors (GMP CD16/32^+^CD34^+/int^) and megakaryocyte-erythroid progenitors (MEP CD16/32^-^CD34^-^) (1, 5, 56). Furthermore, CMPs give rise to the monocyte-dendritic cell progenitor (MDP) compartment (CD135^+^CD117^+^), which contribute together with GMPs to the common monocyte progenitors (cMoP) (CD135^-^Ly6C^+^) that can differentiate into tissue-specific macrophages in case needed (17, 19, 26, 27, 57, 58).

**Figure 3:**
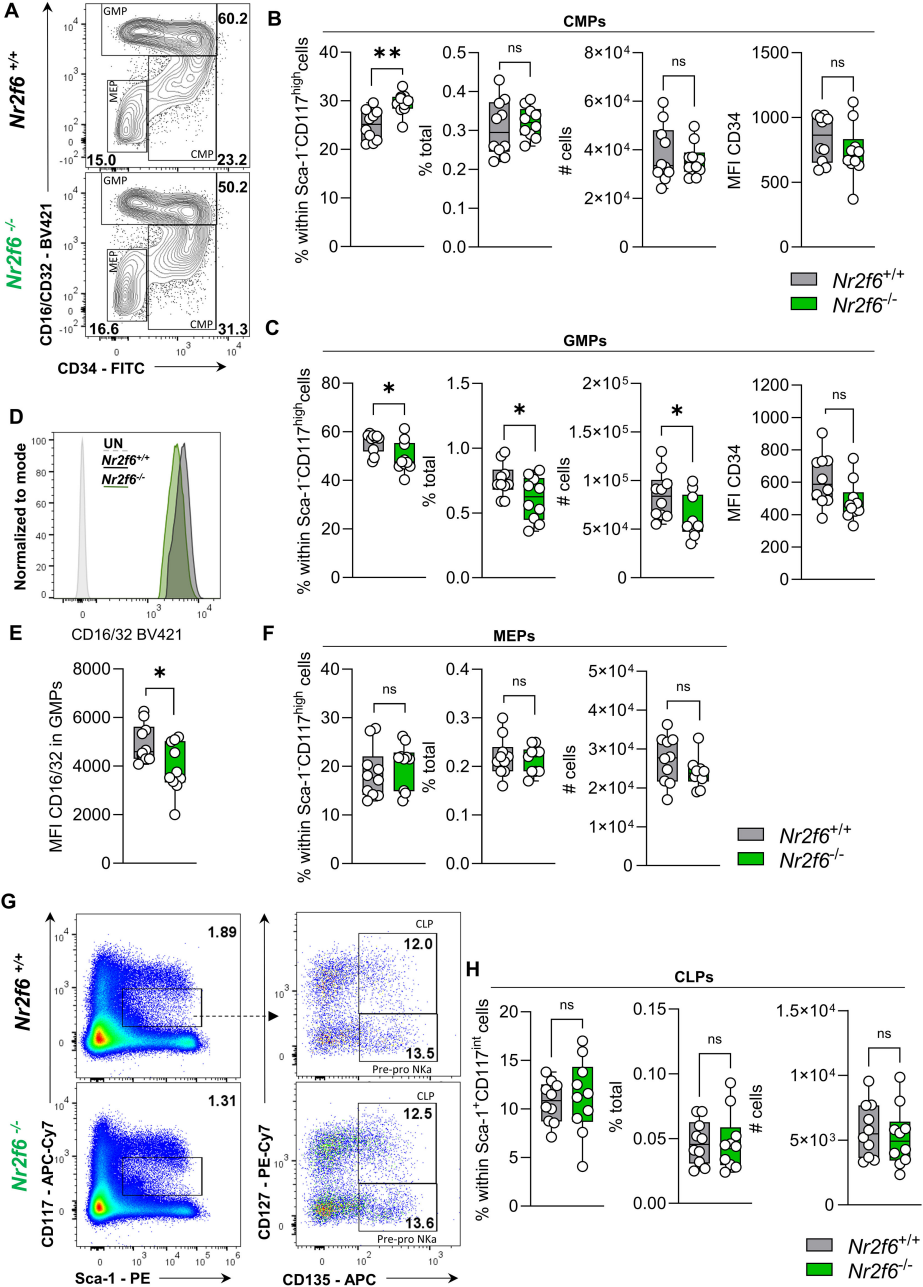
Development of *Nr2f6*-deficient myeloid and lymphoid progenitor populations. **(A)** Representative dot plots of bone marrow-derived live (Lin)^-^CD127^-^Sca-1^-^c-kit (CD117)^+^CD115^-^ CD16/32^+^ (GMP), CD16/32^-^CD34^+^ (CMP), and CD16/32^-^CD34^-^ (MEP) cell populations from wild-type (*Nr2f6* ^+/+^) or *Nr2f6*-deficient (*Nr2f6* ^-/-^) mice. **(B, C)** Quantification of the percent of parent, percent of total, total cell numbers and MFI of CD34 of CD16/32^-^CD34^+^ (CMP) **(B)** and CD16/32^+^ (GMP) **(C)** within live (Lin)^-^CD127^-^ Sca-1^-^c-kit (CD117)^+^CD115^-^ populations from wild-type (*Nr2f6* ^+/+^) or *Nr2f6*-deficient (*Nr2f6* ^-/-^) mice. **(D)** Histogram of the CD16/32 expression within the GMP population from wild-type (*Nr2f6* ^+/+^) or *Nr2f6*-deficient (*Nr2f6* ^-/-^) mice. **(E)** Quantification of the CD16/32 MFI within the GMP population from wild-type (*Nr2f6* ^+/+^) or *Nr2f6*-deficient (*Nr2f6* ^-/-^) mice. **(F)** Quantification of the percent of parent, percent of total and total cell numbers of CD16/32^-^CD34^-^ (MEP) within live (Lin)^−^CD127^-^Sca-1^-^c-kit (CD117)^+^CD115^-^ populations from wild-type (*Nr2f6* ^+/+^) or *Nr2f6*-deficient (*Nr2f6* ^-/-^) mice. **(G)** Representative dot plots of bone marrow-derived live (Lin)^−^ Sca-1^+^c-kit (CD117)^mid^ CD135^+^, CD127^+^ (CLP) and CD127^-^ (pre-pro NKa) cell populations from wild-type (*Nr2f6* ^+/+^) or *Nr2f6*-deficient (*Nr2f6* ^-/-^) mice. **(H)** Quantification of the percent of parent, percent of total and total cell numbers of live (Lin)^−^Sca-1^+^c-kit (CD117)^lo-int^ CD127^+^ (CLP) populations of wild-type (*Nr2f6* ^+/+^) or *Nr2f6*-deficient (*Nr2f6* ^-/-^) mice. Representative data are shown as pooled experiments of at least three independent experiments, total *n* = 10/10 (*Nr2f6^+/+^*)/(*Nr2f6^-/-^*). Each dot represents the data of an individual mouse. Results are shown as median ± IQR with whiskers from min. to max. The Shapiro-Wilk test evaluated the normality of data. Asterisks indicate statistically significant differences between genotypes calculated using the Student’s *t*-test or Mann-Whitney *U* test for non-parametric data. A *p*-value < 0.05 was considered statistically significant, *0.05, **0.01 (See also **Supplementary Figure S3**).

NR2F6 has been shown to inhibit hematopoietic cell differentiation (42). Consequently, we examined the early hematopoietic common myeloid (CMP) and common lymphoid progenitors (CLP) in the bone marrow of wild-type and *Nr2f6*-deficient mice. CMPs, megakaryocyte-erythrocyte progenitors (MEP) and granulocyte-macrophage progenitors (GMP) express high levels of CD117 but not Sca-1 or CD115. The percentage of parent and total as well as total cell numbers of Sca-1^-^CD117^high^CD115^-^ cells were similar between the genotypes (**Supplementary Figure 2E**). Despite an increase in the percentage of parent CMP population in *Nr2f6*-deficient bone marrow, the percentage of total and total cell numbers of CMPs were unaltered compared to wild-type controls (**Figures 3A, B**, **Supplementary Figure 2F**). CD34 (MFI) expression within CMPs was similar between the genotypes (**Figure 3B**).

In contrast, GMPs exhibited a significantly reduced percentage of parent and total cells, with correspondingly decreased total cell numbers in the bone marrow of *Nr2f6*-deficient mice compared to wild-type controls (**Figures 3A, C**). While CD34 expression was unaltered, the MFI of CD16/32 was significantly reduced in the *Nr2f6*-deficient GMP progenitors, potentially contributing to the diminished GMP cell population (**Figures 3C–E**). Conversely, MEP populations were unaltered between the genotypes (**Figures 3A, F**).

We further investigated whether the CD34^high^ or CD34^lo-int^ GMP population contributed to the GMP decrease. Within the GMPs, the percentage of CD34^lo-int^ cells was elevated in *Nr2f6*-deficient compared to control mice (**Supplementary Figure 2G, H**). However, the percentage of total was similar between the genotypes, and cell counts were significantly reduced in the CD34^high^ populations in *Nr2f6*-deficient mice and CD34^lo-int^ GMPs showed a similar trend, suggesting that both cell populations contribute to the decrease in overall GMPs (**Supplementary Figures 2G, I**).

In contrast to expectations, CMPs from *Nr2f6*-deficient bone marrow showed lower proliferation than those from controls. A comparable trend, although not statistically significant, was observed in GMPs, while MEP from both genotypes showed similar Ki-67 staining (**Supplementary Figure 2J**).The percentage of parent and total, as well as the total cell numbers of CLPs in the bone marrow of *Nr2f6*-deficient mice, were similar to wild-type controls (**Figures 3G, H**, **Supplementary Figure 3A**). This gating strategy offered the opportunity to define the natural killer (NK) pre-pro NKa progenitor population (CD135^+^CD127^-^) (59–61), which was unaltered in *Nr2f6*-deficient mice when compared to wild-type (**Figure 3G**, **Supplementary Figures 3A, B**). We also determined the MFI of CD127 and CD135 within the *Nr2f6*-deficient CLP population, which was not altered for CD127 but reduced for CD135 compared to wild-type (**Supplementary Figure 3C**).

### Loss of NR2F6 enhances the MDP progenitor population

To further specify the impact of NR2F6 on myeloid progenitor populations, we conducted a detailed analysis of the monocyte-dendritic cell progenitors (MDP) and common dendritic cell progenitor (CDP) populations via investigating CD117^+^ versus CD117^lo-int^ populations within the Lin^−^CD11b^-^CD115^+^CD135^+^ cell population (26, 27), (**Supplementary Figures 3D, E**).

**Figure 4:**
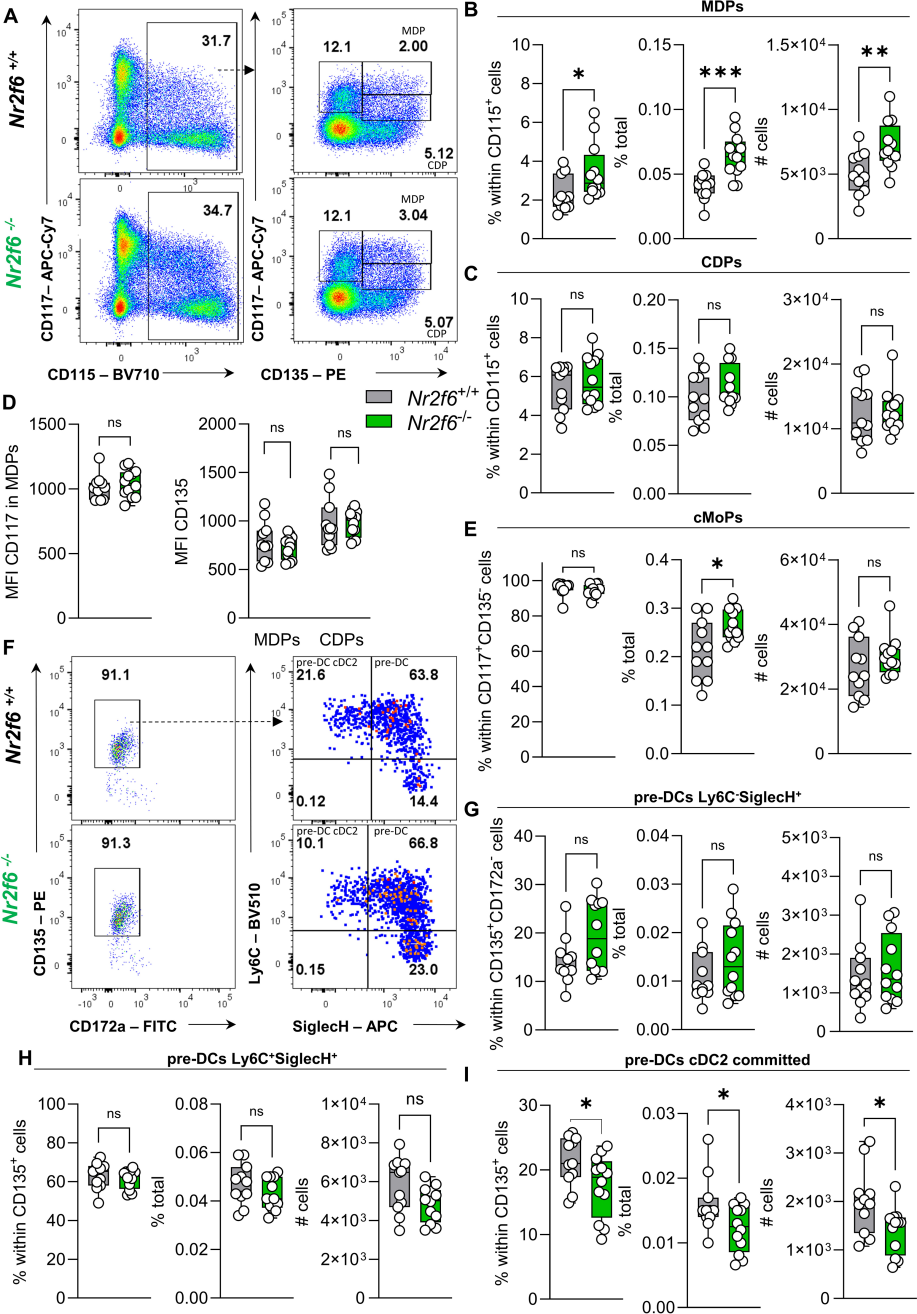
Loss of NR2F6 enhances MDP progenitor populations. **(A)** Representative dot plots of bone marrow-derived live (Lin)^-^CD11b^-^ CD115^+^, c-kit (CD117)^+^CD135^+^ (MDP) and c-kit (CD117)^int^CD135^+^ (CDP) cell populations from wild-type (*Nr2f6* ^+/+^) or *Nr2f6*-deficient (*Nr2f6* ^-/-^) mice. **(B, C)** Quantification of the percent of parent, percent of total and total cell numbers of live (Lin)^-^CD11b^-^CD115^+^c-kit (CD117)^+^CD135^+^ (MDP) **(B)** and c-kit (CD117)^int^CD135^+^ (CDP) **(C)** populations of wild-type (*Nr2f6* ^+/+^) or *Nr2f6*-deficient (*Nr2f6* ^-/-^) mice. **(D)** Quantification of the CD117 MFI within the MDP population and CD135 MFI within MDP and CDP populations from wild-type (*Nr2f6* ^+/+^) or *Nr2f6*-deficient (*Nr2f6* ^-/-^) mice. **(E)** Quantification of the percent of parent, percent of total and total cell numbers of live (Lin)^-^CD11b^-^CD115^+^c-kit (CD117)^+^CD135^-^Ly6C^+^ common monocyte progenitors (cMoP) populations of wild-type (*Nr2f6* ^+/+^) or *Nr2f6*-deficient (*Nr2f6* ^-/-^) mice. **(F)** Representative dot plots of bone marrow-derived live (Lin)^−^CD11b^-^CD11c^+^MHC-II^-^CD135^+^CD172a, Ly6C^+^SiglecH^+^ and Ly6C^-^SiglecH^+^ (pre-DC), as well as Ly6C^+^SiglecH^-^ (pre-DC cDC2) cell populations from wild-type (*Nr2f6* ^+/+^) or *Nr2f6*-deficient (*Nr2f6* ^-/-^) mice. **(G-I)** Quantification of the percent of parent, percent of total and total cell numbers of live (Lin)^−^CD11b^-^CD11c^+^MHC-II^-^CD135^+^CD172a^-^ Ly6C^-^SiglecH^+^ (pre-DC) **(G)** Ly6C^+^SiglecH^+^ (pre-DC) **(H)** and Ly6C^+^SiglecH^-^ (pre-DC cDC2) **(I)** populations of wild-type (*Nr2f6* ^+/+^) or *Nr2f6*-deficient (*Nr2f6* ^-/-^) mice. Representative data are shown as pooled experiments of at least three independent experiments, total *n* = 11/12 (*Nr2f6^+/+^*)/(*Nr2f6^-/-^*). Each dot represents the data of an individual mouse. Results are shown as median ± IQR with whiskers from min. to max. The Shapiro-Wilk test evaluated the normality of data. Asterisks indicate statistically significant differences between genotypes calculated using the Student’s *t*-test or Mann-Whitney *U* test for non-parametric data. A *p*-value < 0.05 was considered statistically significant, *0.05, **0.01, ***0.001 (See also **Supplementary Figures S3** and **S4**).

In *Nr2f6*-deficient mice, the percentages of parent, total, and total CD135^+^CD117^+^ MDP cell numbers were significantly enhanced (**Figures 4A, B**). In contrast, we observed no difference in the percentages of parent, total, or total cell numbers of CD135^+^CD117^lo-int^ CDPs in *Nr2f6*-deficient mice when compared to wild-type (**Figure 4C**). The MFI of CD117 and CD135 were similar within both genotypes (**Figure 4D**).

Analyzing the common monocyte progenitors (cMoP) within the Lin^−^CD11b^-^CD115^+^ CD117^+^CD135^-^ cell population (**Supplementary Figures 3D–F**), we found no differences in percentages of parent or total cell numbers despite enhanced percentage of total cells in *Nr2f6*-deficient mice (**Figure 4E**). The same pattern was observed in the pre-gate within the CD135CD117^+^ population (**Supplementary Figure 3G**). Ki-67 staining in MDPs, CDPs, and cMoPs was comparable between genotypes (**Supplementary Figure 3H**).

### Pre-DCs committed to the cDC2 lineage are reduced in *Nr2f6*-deficient bone marrow

MDPs are also able to generate common dendritic cell (DC) progenitors (CDP) (CD135^+^CD117^-^), which produces pre-conventional (c)DCs and plasmacytoid (p)DCs (23, 27, 28).

To investigate potential alterations beyond the CDP population, we assessed pre-DCs with potential for conventional (c)DC differentiation (Lin^−^CD11b^-^CD11c^+^MHC-II^-^CD135^+^CD172a^-^ Ly6C^+^SiglecH^+^ & Ly6C^-^SiglecH^+^) as well as pre-DCs committed to the cDC1 lineage (CD135^+^CD11c^+^CD117^int^/CD226^+^ Ly6C^−^SiglecH^−^) and pre-DCs committed to the cDC2 lineage (Lin^−^CD11b^-^CD11c^+^MHC-II^-^CD135^+^CD172a^-^ Ly6C^+^SiglecH^−^) (23, 24, 27). (**Supplementary Figures 4A, B**). Analysis of *Nr2f6*-deficient BM revealed no alterations in percentages of parent, total and total cell numbers in the CD135^+^CD11c^+^ population, as well as the pre-cDC1 progenitors compared to wild-type controls (**Supplementary Figures 4B–D**). The Ly6C^−^SiglecH^+^ and Ly6C^+^SiglecH^+^ pre-DCs precursor populations were also unaltered between the genotypes (**Figures 4F–H**). However, the *Nr2f6*-deficient Ly6C^+^SiglecH^−^ population (pre-DCs committed to the cDC2 lineage) was significantly reduced in both percentages of parent and total as well as total cell numbers (**Figures 4F, I**). Despite these changes, the proliferative capacities of pre-DCs committed to either the cDC1 or cDC2 lineage were comparable between genotypes (**Supplementary Figure 4E**).

In conclusion, the loss of NR2F6 reduced the GMP but not the CMP or CLP progenitor population. In contrast, the MDP progenitors but not CDP and cMoP progenitor populations were increased in *Nr2f6*-deficient mice. In addition, the loss of NR2F6 reduced the pre-cDC2 but not the pre-cDC1 population (**Supplementary Figure 4F**).

### *Nr2f6*-deficiency affects myelopoiesis independent of the bone marrow microenvironment

**Figure 5:**
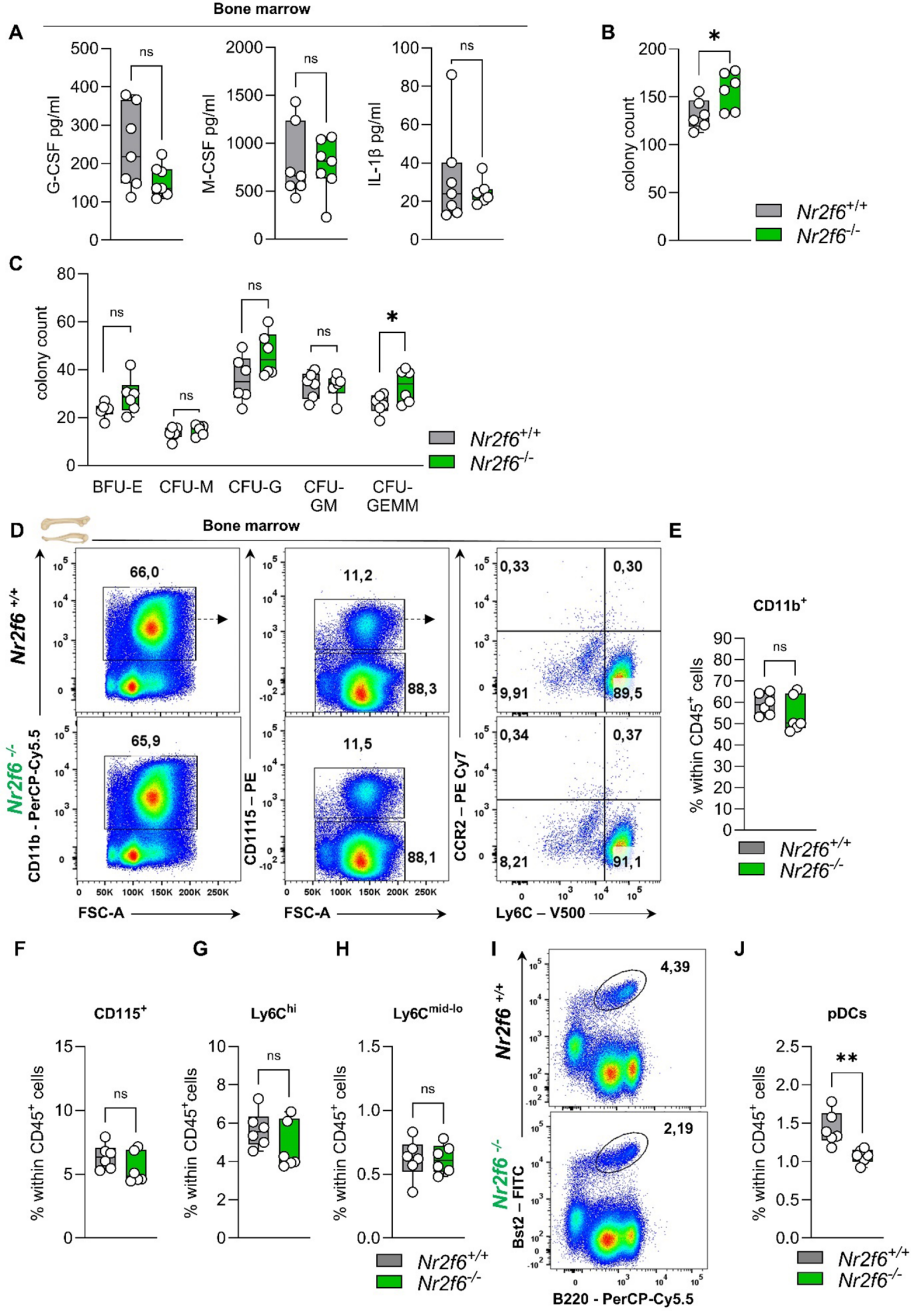
Cytokines, CFUs, and the myeloid compartment in the bone marrow of *Nr2f6*-deficient mice. **(A)** Quantification of G-CSF, M-CSF, and IL-1β concentrations (in pg/ml) in the bone marrow supernatant of wild-type (*Nr2f6* ^+/+^) or *Nr2f6*-deficient (*Nr2f6* ^-/-^) mice. **(B, C)** Quantification of the total CFU count **(B)** as well as specific colonies **(C)**, derived from the bone marrow in mouse methylcellulose complete media eight days post seeding of wild-type (*Nr2f6* ^+/+^) or *Nr2f6*-deficient (*Nr2f6* ^-/-^) mice. **(D)** Representative dot plots of bone marrow-derived CD45^+^CD11b^+^, CD115^+^, CD115^-^, Ly6C^hi^ and Ly6C^mid-lo^ cell populations from wild-type (*Nr2f6* ^+/+^) or *Nr2f6*-deficient (*Nr2f6* ^-/-^) mice. **(E-H)** Quantification of bone marrow-derived CD11b^+^ **(E)**, CD115^+^ **(F)**, Ly6C^hi^ **(G)** and Ly6C^mid-lo^ **(H)** cell populations within CD45^+^ cells from wild-type (*Nr2f6* ^+/+^) or *Nr2f6*-deficient (*Nr2f6* ^-/-^) mice. **(I-J)** Representative dot plots **(I)** and quantification **(J)** of bone marrow-derived Bst2^+^B220^+^ pDCs within CD45^+^ cells from wild-type (*Nr2f6* ^+/+^) or *Nr2f6*-deficient (*Nr2f6* ^-/-^) mice. Representative data are shown as pooled experiments of two independent experiments, total *n* = 6/6 (*Nr2f6^+/+^*)/(*Nr2f6^-/-^*) per genotype. Each dot represents the data of an individual mouse. Results are shown as median ± IQR with whiskers from min. to max. The Shapiro-Wilk test evaluated the normality of data. Outliers were excluded using ROUT (Q=1%) in GraphPad Prism. Asterisks indicate statistically significant differences between genotypes calculated using the nested *t*-test **(B, C),** Student’s *t*-test, or Mann-Whitney *U* test for non-parametric data. A *p*-value < 0.05 was considered statistically significant, *0.05, **0.01, (See also **Supplementary Figure S5**).

To investigate potential changes in the bone marrow microenvironment, we measured cytokine concentrations of G-CSF, M-CSF, and IL-1β in the bone marrow supernatant. While there was a slight trend towards reduced G-CSF levels, no significant differences in M-CSF and IL-1β concentrations were observed between wild-type and *Nr2f6*-deficient mice (**Figure 5A**).

To determine how Nr2f6 affects hematopoiesis independently of the bone marrow environment, an *in vitro* colony-forming unit (CFU) assay was performed. The cells can differentiate into burst-forming units-erythroid (BFU-E), colony-forming unit-granulocytes (CFU-G), colony-forming unit-macrophages (CFU-M), colony-forming unit-granulocyte, macrophage (CFU-GM) and colony forming unit-granulocyte, erythrocyte, macrophage, megakaryocyte (CFU-GEMM). CFU-G are clonogenic progenitors of granulocytes that give rise to a homogeneous population of eosinophils, basophils, or neutrophils, while CFU-M are clonogenic progenitors of macrophages that give rise to a homogenous population of macrophages. CFU-GM arise from earlier progenitors that give rise to colonies containing a heterogeneous population of macrophages and granulocytes. CFU-GEMM originates from multi-lineage progenitors that give rise to erythroid, granulocytes, macrophages, and megakaryocyte lineage.

The bone marrow of *Nr2f6*-deficient mice exhibited an unexpected increase in the total number of colonies that formed (**Figure 5B**). However, the number of BFU-E, CFU-M and CFU-GM did not vary between the genotypes. The observed differences were attributed to a significant increase in CFU-GEMM, as well as a notable, although not statistically significant, increase in CFU-G (**Figure 5C**).

### Bone marrow, blood and splenic myeloid cell populations in *Nr2f6*-deficient mice

As a next step, we investigated how alterations in hematopoiesis and myelopoiesis influence the myeloid compartment in the bone marrow, blood, and spleen and of adult *Nr2f6*-deficient mice.

In the bone marrow, the percentages of parent and of total CD45^+^ CD11b^+^ myeloid cells, CD115^+^ monocytes, and both classical Ly6C^hi^ and non-classical Ly6C^mid-lo^ monocytes were comparable between genotypes (**Figures 5D–H**, **Supplementary Figure 5A**). The percent of parent Ly6G^+^ neutrophils was enhanced, but no alterations were observed within the CD45^+^ cell population (**Supplementary Figures 5A, B**). Notably, the Bst2^+^B220^+^ plasmacytoid dendritic cells (pDCs) percentage of parent and CD45^+^ in the bone marrow was reduced (**Figure 5I, J**, **Supplementary Figure 5A**).

**Figure 6:**
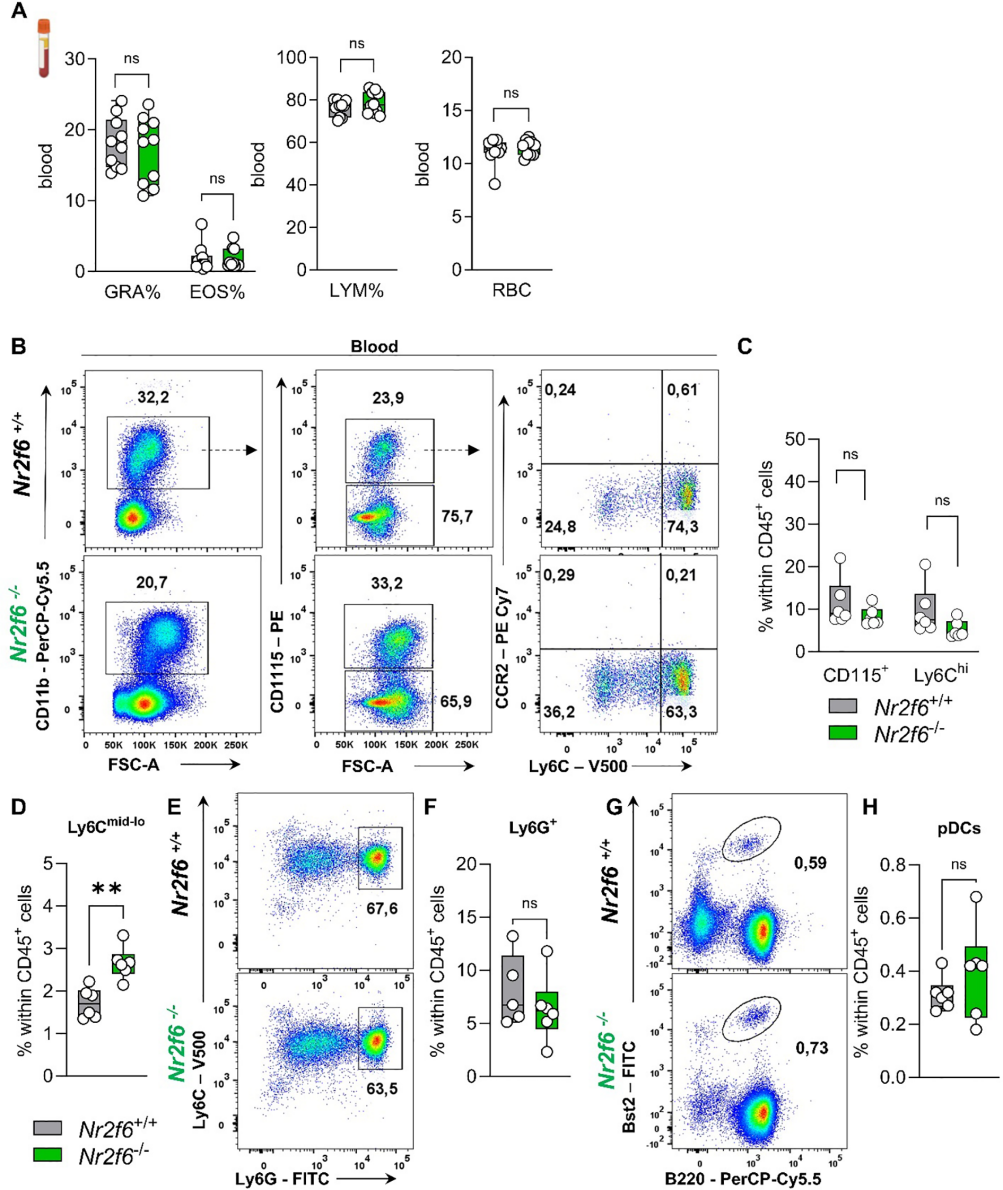
Myeloid compartment in the blood of *Nr2f6*-deficient mice. **(A)** Quantification of the percent of granulocytes (GRA%), eosinophils (EOS%) and lymphocytes (LYM) as well as the percent of red blood cells (RBC) in the blood in wild-type (*Nr2f6* ^+/+^) or *Nr2f6*-deficient (*Nr2f6* ^-/-^) mice. **(B)** Representative dot plots of blood-derived CD45^+^CD11b^+^, CD115^+^, CD115^-^, Ly6C^hi^ and Ly6C^mid-lo^ cell populations from wild-type (*Nr2f6* ^+/+^) or *Nr2f6*-deficient (*Nr2f6* ^-/-^) mice. **(C, D)** Quantification of blood-derived CD115^+^ **(C)**, Ly6C^hi^ **(C)** and Ly6C^mid-lo^ **(D)** cell population within CD45^+^ cells from wild-type (*Nr2f6* ^+/+^) or *Nr2f6*-deficient (*Nr2f6* ^-/-^) mice. **(E, F)** Representative dot plots **(E)** and quantification **(F)** of blood-derived CD115^-^Ly6C^hi^Ly6G^hi^ neutrophils within CD45^+^ cells from wild-type (*Nr2f6* ^+/+^) or *Nr2f6*-deficient (*Nr2f6* ^-/-^) mice. **(G, H)** Representative dot plots **(G)** and quantification **(H)** of blood-derived Bst2^+^B220^+^ pDCs within CD45^+^ cells from wild-type (*Nr2f6* ^+/+^) or *Nr2f6*-deficient (*Nr2f6* ^-/-^) mice. Representative data are shown as pooled experiments of two independent experiments, total *n* = 6-10/6-10 (*Nr2f6^+/+^*)/(*Nr2f6^-/-^*) per genotype. Each dot represents the data of an individual mouse. Results are shown as median ± IQR with whiskers from min. to max. The Shapiro-Wilk test evaluated the normality of data. Outliers were excluded using ROUT (Q=1%) in GraphPad Prism. Asterisks indicate statistically significant differences between genotypes calculated using the nested *t*-test **(B, C),** Student’s *t*-test, or Mann-Whitney *U* test for non-parametric data. A *p*-value < 0.05 was considered statistically significant, **0.01 (See also **Supplementary Figure S5**).

In the blood, the proportions of granulocytes, eosinophils, lymphocytes, or red blood cells were similar between the genotypes using a Vet ABC ™Hematology Analyzer (**Figure 6A**).

In more detail, we investigated CD115^+^ monocytes, including classical Ly6C^hi^ and non-classical Ly6C^mid-lo^ monocytes, as well as Ly6G^+^ neutrophils and Bst2^+^B220^+^ pDCs in the blood of wild-type and *Nr2f6*-deficient mice (**Supplementary Figure 5C**). The percentage of total monocytes (CD115^+^) within the CD45^+^ cell population remained unaltered (**Figures 6B, C**), but the ratio of Ly6C^hi^ to Ly6C^mid-lo^ was significantly altered (**Supplementary Figure 5D**), resulting in enhanced non-classical monocytes within CD45^+^ cells when compared to wild-type (**Figure 6D**). We investigated CCR2 expression to detect inflammatory Ly6C^hi^CCR2^+^ monocytes but could not detect this subset during steady-state in either genotype (**Figure 6B**). Analysis of Ly6G^+^ neutrophils and Bst2^+^B220^+^ pDCs revealed no differences in the percent of parent and of CD45^+^ cells between genotypes (**Figures 6E–H**, **Supplementary Figure 5D**).

**Figure 7:**
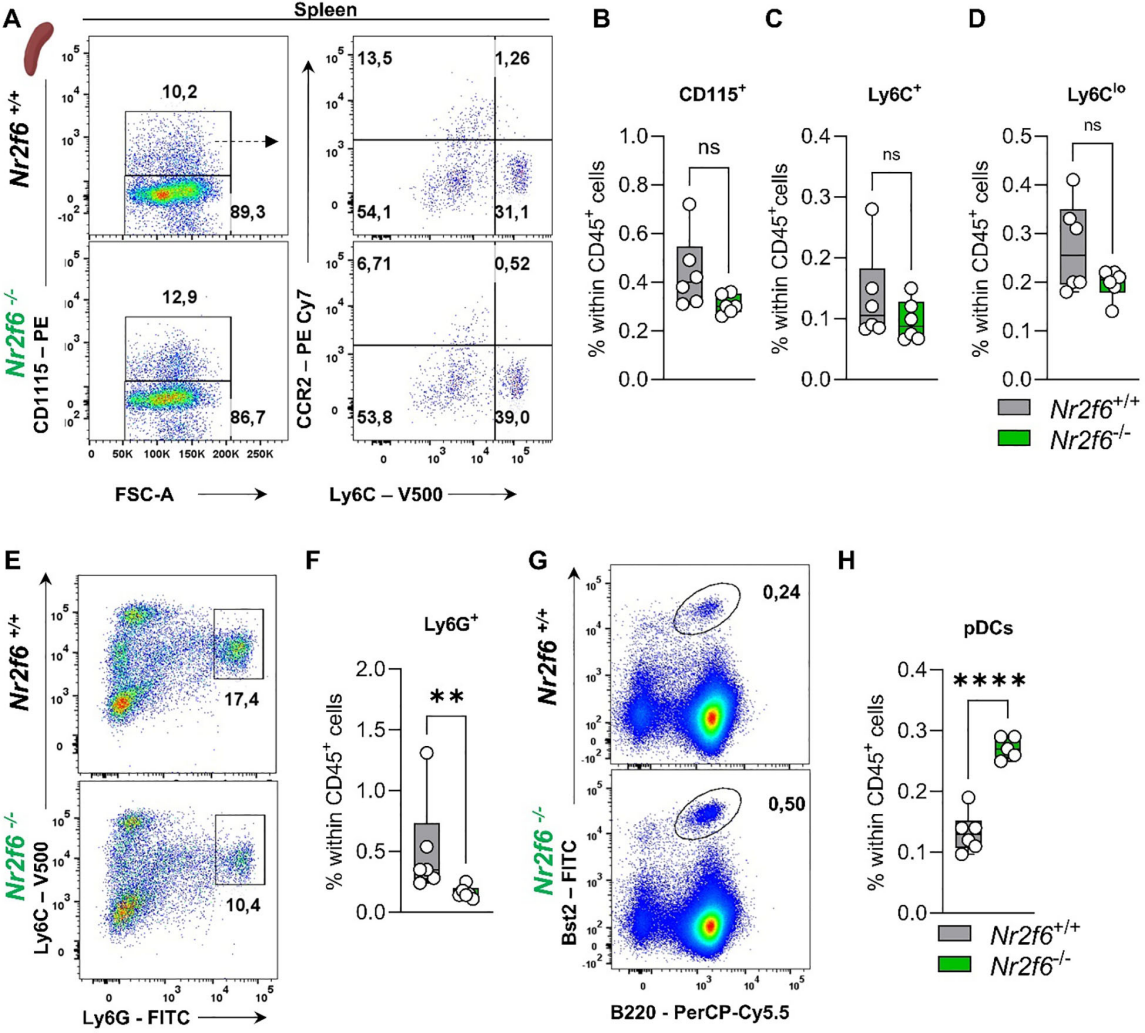
Myeloid compartment in the spleen of *Nr2f6*-deficient mice. **(A)** Representative dot plots of splenic CD115^+^, CD115^-^, Ly6C^hi^ and Ly6C^mid-lo^ cell populations from wild-type (*Nr2f6* ^+/+^) or *Nr2f6*-deficient (*Nr2f6* ^-/-^) mice. **(B-D)** Quantification of CD115^+^ **(B)**, Ly6C^hi^ **(C)**, and Ly6C^mid-lo^ **(D)** cells within splenic CD45^+^ cells of wild-type (*Nr2f6* ^+/+^) or *Nr2f6*-deficient (*Nr2f6* ^-/-^) mice. **(E, F)** Representative dot plots **(E)** and quantification **(F)** of splenic CD115^-^Ly6C^hi^Ly6G^hi^ cell populations within splenic CD45^+^ from wild-type (*Nr2f6* ^+/+^) or *Nr2f6*-deficient (*Nr2f6* ^-/-^) mice. **(G, H)** Representative dot plots **(G)** and quantification **(H)** of splenic Bst2^+^B220^+^ pDC cell populations within splenic CD45^+^ from wild-type (*Nr2f6* ^+/+^) or *Nr2f6*-deficient (*Nr2f6* ^-/-^) mice. Representative data are shown as pooled experiments of two independent experiments, total *n* = 6/6 (*Nr2f6^+/+^*)/(*Nr2f6^-/-^*). Each dot represents the data of an individual mouse. Results are shown as median ± IQR with whiskers from min. to max. The Shapiro-Wilk test evaluated the normality of data. Outliers were excluded using ROUT (Q=1%) in GraphPad Prism. Asterisks indicate statistically significant differences between genotypes calculated using the Student’s *t*-test or Mann-Whitney *U* test for non-parametric data. A *p*-value < 0.05 was considered statistically significant, **0.01, ****0.0001 (See also **Supplementary Figure S5**).

In the spleen of *Nr2f6*-deficient mice, the percentages of total monocytes, as well as Ly6C^hi^ and Ly6C^mid-lo^ monocytes, were similar to those of the controls (**Figures 7A–D**, **Supplementary Figure 5E**). However, Ly6G^+^ neutrophils were reduced, while Bst2^+^B220^+^ pDCs were elevated compared to the controls (**Figures 7E–H**, **Supplementary Figure 5E**). Details on dendritic and macrophage populations in the spleen of *Nr2f6*-deficient mice are currently under revision elsewhere (Woelk-J et al. and Benz-J et al.).

In summary, *Nr2f6*-deficiency results in reduced pDCs in the bone marrow and enhanced non-classical monocytes in the blood. In the spleen, reduced neutrophils but increased pDC populations were detected in *Nr2f6*-deficient mice.

## Discussion

We investigated hematopoietic stem cell (HSCs) dynamics and hematopoiesis in germline *Nr2f6*-deficient bone marrow. Previously, the nuclear receptor NR2F6 (EAR-2) has been shown to regulate murine hematopoietic cell differentiation, and overexpression induces myeloid dysplasia in a retroviral transfection model (41, 42). Along this line, Ichim et al. have shown that shRNA silencing of NR2F6 (EAR-2) results in a loss of the stem cell fraction, inhibition of self-renewal combined with lower proliferative capacity and a concurrent increase in cells expressing myeloid markers *in vitro* (42).

Nonetheless, the methods used by Ichim et al., including chimeras, lentiviral transduction, 5-FU treatment, and sorting strategies, differ from the germline-deficient mouse model used in this study, each with its own limitations. These methodological differences complicate direct comparisons of the study results.

In the study of Ichim et al., NR2F6 (EAR-2) transduced bone marrow cells maintain an immature immunophenotype, while silencing of NR2F6 (EAR-2) expression with shRNA has been shown to cause a selective loss of the stem cell fraction, inhibition of self-renewal and lower proliferative capacity (42). Although proliferative capacity (Ki-67) was reduced in the stem cell fraction, in our germline *Nr2f6*-deficient mouse model, we could not detect a decrease in any hematopoietic stem cell population, but an increase in LSK^+^ due to higher numbers of LT&ST-HSCs as well as the MPP3 populations.

Furthermore, Ichim et al. showed that NR2F6 (EAR-2) knockdown induced granulocytic differentiation (42), which is along the line that we could observe increased populations of CFU-GEMM in our colony forming assays. In contrast, our study showed that the percentage of total GMP and pre-cDC2 were significantly reduced in *Nr2f6*-deficient mice compared to wild-type controls. *In vitro*, CFU assays showed an increase in the total number of colonies that formed, accounted for by an increase in CFU-GEMM colonies in the absence of *Nr2f6*. We speculate that the above discrepancies are caused by the complexity of the bone marrow microenvironment, including leptin-receptor-positive mesenchymal stromal cells, vascular endothelial cells, osteoblasts, sympathetic nerve fibers, macrophages, megakaryocytes, and non-myelinating Schwann cells, all of which contribute signals to the hematopoietic stem cell niche might be altered in the germline *Nr2f6*-deficient mouse model.

We have previously shown that *Nr2f6*-deficient mice develop a late-onset auto-immunopathology on a 129SvEv^Brd^ C57BL/6J hybrid background (43). Furthermore, loss of NR2F6 alters intestinal permeability and results in spontaneous late-onset colitis (50). Both intestinal dysbiosis and the IL-23/Th17 pathway have been shown to influence hematopoiesis in the bone marrow and can increase, in parallel to our findings in *Nr2f6*-deficient mice, the LSK^+^ and LT&ST-HSCs and MPP3 populations (62, 63). However, in contrast to inflammatory settings, GMP progenitors are not enhanced but reduced in *Nr2f6*-deficient mice, suggesting a different cause. Along this line, we did not detect differences in the cytokine concentrations of G-CSF, M-CSF, and IL-1β in bone marrow supernatant.

NRs are well-known regulators of HSC differentiation (31, 33). For instance, antagonism of the retinoic acid receptor (RAR) impacts mouse HSC reconstituting ability while supporting human HSC maintenance (35, 36, 38). In contrast to the bone marrow of *Nr2f6*-deficient mice, RARγ knockout mice exhibit markedly reduced numbers of HSCs associated with increased numbers of more mature progenitor cells (38). Furthermore, the RAR/RXR heterodimer is a critical regulator of human HSC differentiation, and pharmacological modulation of RXR signaling prevents the loss of human HSCs that otherwise occurs in short-term culture (39). Because RXR can function as a homodimer or as a heterodimer with other nuclear receptors (NR) such as NR2F6, it is unclear whether the data of this study on hematopoietic progenitors occurs through the same pathway or whether they involve the regulation of parallel pathways.

In *Nr4a3^−/−^Nr4a1^−/−^* bone marrow, LT-HSCs were expanded, in parallel to *Nr2f6*-deficient LT-HSCs percentage of total, but short-term HSCs (ST-HSCs) were unaltered (37). In contrast to the enhanced *Nr2f6*-deficient LSK population, this population was not affected by the loss of *Nr4a3^−/−^Nr4a1^−/−^* but an abnormal expansion of myeloid progenitors could be observed in the latter ones (37).

If and how NR2F6 interacts with these other NRs in specific hematopoietic stem cell progenitor populations await future investigations.

Analysis of how the alterations in *Nr2f6*-deficient mice during hematopoiesis and myelopoiesis influence the myeloid compartment in the periphery of *Nr2f6*-deficient mice revealed altered non-classical monocytic population in the blood and reduced neutrophils in the spleen. The observation that pDC numbers were reduced in the bone marrow but enhanced in the spleen needs further investigation.

In addition, analysis of NR2F6 regulated process during emergency granulopoiesis in infection or tumor models will be of interest in future studies. To define the transcriptional landscape and mechanistic role of NR2F6 in defined bone marrow populations and peripheral myeloid immune cells in detail, independent of the non-immune microenvironment, it will be mandatory to investigate hematopoiesis in an immune cell-specific inducible mouse knockout model in the future.

## Data Availability

The raw data supporting the conclusions of this article will be made available by the authors, without undue reservation.
