# Supplementary material for: NR2F6 regulates stem cell hematopoiesis and myelopoiesis in mice
Source: Front Immunol. 2025 Jan 7;15:1404805. doi: 10.3389/fimmu.2024.1404805 (PMC11747239; doi:10.3389/fimmu.2024.1404805)
Supplement: Supplementary file 1 [file DataSheet1.pdf]

Supplementary Material

NR2F6 regulates stem cell hematopoiesis and myelopoiesis in mice

Johannes Woelk[1], Hamsa Narasimhan[2, 3], Christa Pfeifhofer-Obermair[4], Barbara U Schraml[2, 3], and Natascha Hermann-Kleiter[1*]

[1]Institute of Cell Genetics, Department for Genetics and Pharmacology, Medical University of Innsbruck, Innsbruck, Austria.

[2]Institute for Immunology, Faculty of Medicine, LMU Munich, Munich, Germany.

[3]Institute of Cardiovascular Physiology and Pathophysiology at the Walter-Brendel-Centre of Experimental Medicine, Faculty of Medicine, LMU Munich, Munich, Germany.

[4]Department of Internal Medicine II (Infectious Diseases, Immunology, Rheumatology, Pneumology), Medical University of Innsbruck, Innsbruck, Austria.

*Correspondence: natascha.kleiter@i-med.ac.at (N-HK)

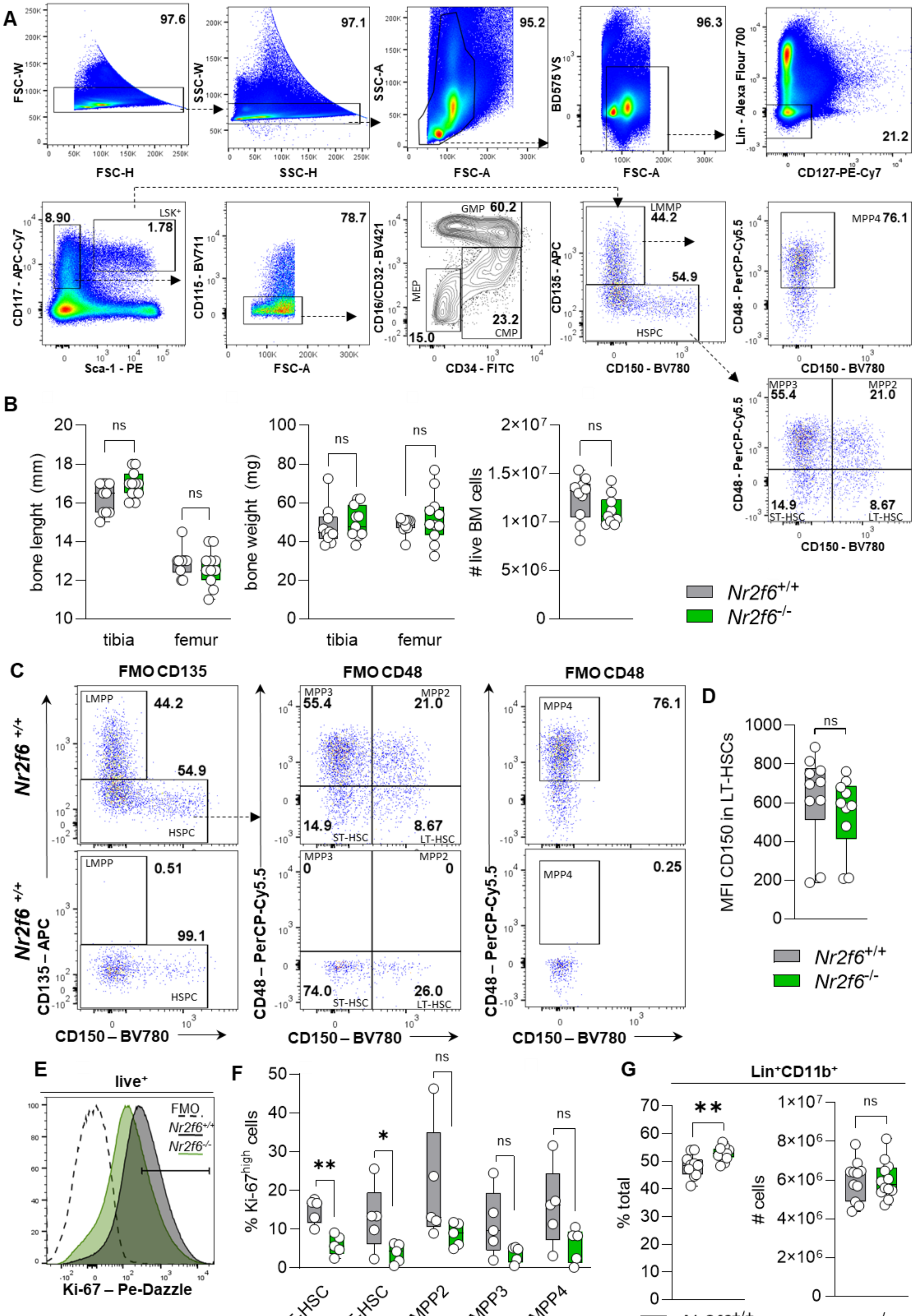

Supplementary Figure 1

Supplementary Figure 1: Gating scheme and FMO controls of hematopoietic stem cell and myeloid progenitor populations.

(A) Representative dot plots of the gating scheme of bone marrow-derived hematopoietic stem cell and myeloid progenitor populations.

(B) Quantification of length (mm) and weight (mg) of the femur or tibia as well as the total bone marrow counts from wild-type (*Nr2f6*$^{+/+}$) or *Nr2f6*-deficient (*Nr2f6*$^{-/-}$) mice.

(C) Representative dot plots of bone marrow-derived live (Lin)$^{-}$CD127^{-} LSK^{+}CD135^{+}CD150^{-} (LMPP), CD135^{-} (HSPC), CD48-CD150^{+} (LT-HSC), CD48^{-}CD150^{-} (ST-HSC), CD48^{+}CD150^{+} (MPP2), CD48^{+}CD150^{-} (MPP3) and LMPP derived CD48^{+}CD150^{-} (MPP4) populations from wild-type (*Nr2f6*$^{+/+}$) mice with FMO controls for CD135 and CD48.

(D) Quantification of the CD150 MFI within LT-HSCs from wild-type (*Nr2f6*$^{+/+}$) or *Nr2f6*-deficient (*Nr2f6*$^{-/-}$) mice.

(E-F) Representative histogram **(E)** and quantification **(F)** of the percent Ki-67^{high} LT-HSC, ST-HSC, MPP2, MPP3 and MPP4 cell populations from bone marrow of wild-type (*Nr2f6*$^{+/+}$) or *Nr2f6*-deficient (*Nr2f6*$^{-/-}$) mice.

(G) Quantification of the percent of total and total cell numbers of live (Lin)$^{+}$CD11b^{+} cell populations from wild-type (*Nr2f6*$^{+/+}$) or *Nr2f6*-deficient (*Nr2f6*$^{-/-}$) mice.

Representative data are shown as pooled experiments of at least two independent experiments, total n =6-10/6-10 (Nr2f6$^{+/+}$)/(*Nr2f6*$^{-/-}$). Each dot represents the data of an individual mouse. Results are shown median ± IQR with whiskers from min. to max. Outliers were excluded using ROUT (Q=1%) in GraphPad Prism. The Shapiro-Wilk test evaluated the normality of data. Asterisks indicate statistically significant differences between genotypes calculated using the Student's *t*-test, or Mann-Whitney *U* test for non-parametric data. A p-value < 0.05 was considered statistically significant, *0.05, **0.01

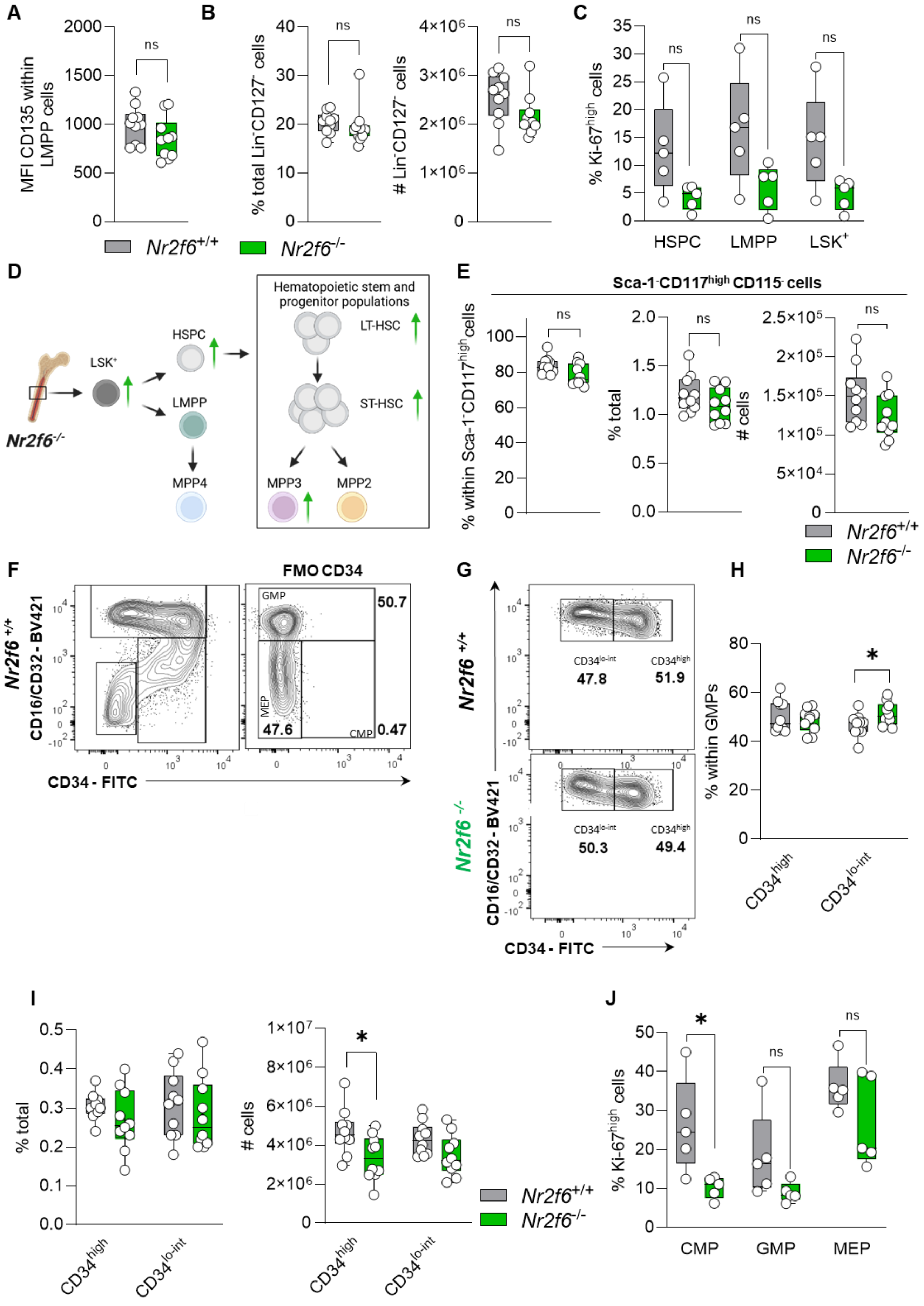

Supplementary Figure 2

Supplementary Figure 2: Quantification of Lin$^-$CD127$^-$, Sca-1$^-$CD117^{high}CD115$^-$ cells and scheme for HSC development in *Nr2f6*-deficient mice.

(A) Quantification of the CD135 MFI within LMPP populations from wild-type (*Nr2f6*$^{+/+}$) or *Nr2f6*-deficient (*Nr2f6*$^{-/-}$) mice.

(B) Quantification of the percent of total and total cell numbers of live (Lin)$^-$CD127$^-$ populations from wild-type (*Nr2f6*$^{+/+}$) or *Nr2f6*-deficient (*Nr2f6*$^{-/-}$) mice.

(C) Quantification of the percent Ki-67$^+$ HSPC, LMPP and LSK$^+$ cell populations from bone marrow of wild-type (*Nr2f6*$^{+/+}$) or *Nr2f6*-deficient (*Nr2f6*$^{-/-}$) mice.

(D) Schematic representation of hematopoietic stem cell (HSCs) differentiation with long-term (LT)-HSC and short-term (ST)-HSC subpopulations and the lineage-biased multipotent progenitor (MPPs) in the bone marrow. Scheme modified after Dress ([27]). Created with BioRender.com.

(E) Quantification of the percent of parent, percent of total and total cell numbers of Sca-1$^-$CD117^{high} CD115$^-$ populations from the bone marrow of wild-type (*Nr2f6*$^{+/+}$) or *Nr2f6*-deficient (*Nr2f6*$^{-/-}$) mice.

(F) Representative dot plots of bone marrow-derived GMP, CMP, and MEP cell populations from wild-type (*Nr2f6*$^{+/+}$) mice and FMO control of CD34.

(G) Representative dot plots of CD34^{high} and CD34$^{lo\text{-}int}$ GMPs from wild-type (*Nr2f6*$^{+/+}$) or *Nr2f6*-deficient (*Nr2f6*$^{-/-}$) mice.

(H) Quantification of the percent of parent of CD34^{high} and CD34$^{lo\text{-}int}$ GMP from wild-type (*Nr2f6*$^{+/+}$) or *Nr2f6*-deficient (*Nr2f6*$^{-/-}$) mice

(I) Quantification of the percent of total and total cell numbers of CD34^{high} and CD34$^{lo\text{-}int}$ GMP from wild-type (*Nr2f6*$^{+/+}$) or *Nr2f6*-deficient (*Nr2f6*$^{-/-}$) mice

(J) Quantification of the percent Ki-67^{high} CMP, GMP, MEP cell population from bone marrow of wild-type (*Nr2f6*$^{+/+}$) or *Nr2f6*-deficient (*Nr2f6*$^{-/-}$) mice.

Representative data are shown as pooled experiments of at least two independent experiments, total n = 6-10/6-10 (*Nr2f6*$^{+/+}$)/(*Nr2f6*$^{-/-}$). Each dot represents the data of an individual mouse. Results are shown median ± IQR with whiskers from min. to max. Outliers were excluded using ROUT (Q=1%) in GraphPad Prism. The Shapiro-Wilk test evaluated the normality of data. Asterisks indicate statistically significant differences between genotypes calculated using the Student's *t*-test, or Mann-Whitney *U* test for non-parametric data. A p-value < 0.05 was considered statistically significant, *0.05.

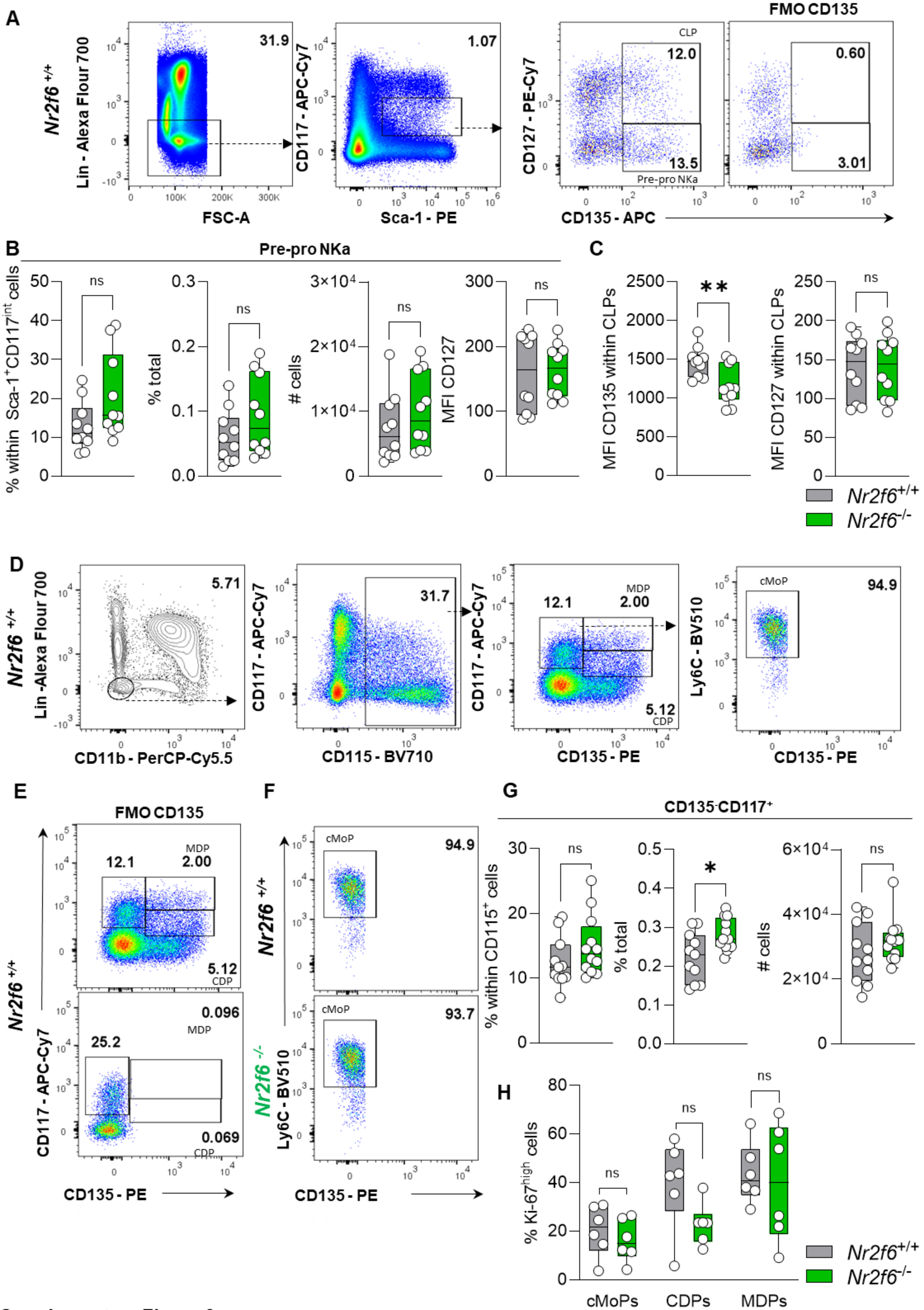

Supplementary Figure 3

Supplementary Figure 3: Development of *Nr2f6*-deficient lymphoid and monocyte progenitor populations.

(A) Representative dot plots of the bone marrow-derived CLP and pre-pro NKa cell populations from wild-type (*Nr2f6*$^{+/+}$) mice and FMO control of CD135.

(B) Quantification of the percent of parent, percent of total, total cell numbers and the MFI of CD127 of bone marrow-derived live (Lin)$^{-}$c-kit(CD117)^{int}Sca-1^{+}, CD127^{-} (pre-pro NKa) populations from wild-type (*Nr2f6*$^{+/+}$) or *Nr2f6*-deficient (*Nr2f6*$^{-/-}$) mice.

(C) Quantification of the CD135 and CD127 MFI in the CLP population from wild-type (*Nr2f6*$^{+/+}$) or *Nr2f6*-deficient (*Nr2f6*$^{-/-}$) mice.

(D) Representative dot plots of the gating scheme of bone marrow-derived MDP, CDP, and cMoP populations.

(E) Representative dot plots of the bone marrow-derived MDP and CDP populations from wild-type (*Nr2f6*$^{+/+}$) mice and FMO control of CD135.

(F) Representative dot plots of bone marrow-derived live (Lin)$^{-}$CD11b^{-}CD115^{+} c-kit(CD117)$^{+}$CD135^{-}Ly6C^{+} (cMoP) cell populations of wild-type (*Nr2f6*$^{+/+}$) or *Nr2f6*-deficient (*Nr2f6*$^{-/-}$) mice.

(G) Quantification of the percent of parent, percent of total and total cell numbers of live (Lin)$^{-}$CD11b^{-}CD115^{+} c-kit(CD117)$^{+}$CD135^{-} population of wild-type (*Nr2f6*$^{+/+}$) or *Nr2f6*-deficient (*Nr2f6*$^{-/-}$) mice.

(H) Quantification of the percent Ki-67^{high} cMoP, CDP, MDP cell populations from bone marrow of wild-type (*Nr2f6*$^{+/+}$) or *Nr2f6*-deficient (*Nr2f6*$^{-/-}$) mice.

Representative data are shown as pooled experiments of at least two independent experiments, total n = 6-12/6-12 (*Nr2f6*$^{+/+}$)/(*Nr2f6*$^{-/-}$). Each dot represents the data of an individual mouse. Results are shown median ± IQR with whiskers from min. to max. The Shapiro-Wilk test evaluated the normality of data. Asterisks indicate statistically significant differences between genotypes calculated using the Mann-Whitney *U*, Student's or Welch's corrected t-test. A p-value < 0.05 was considered statistically significant, *0.05, **0.01.

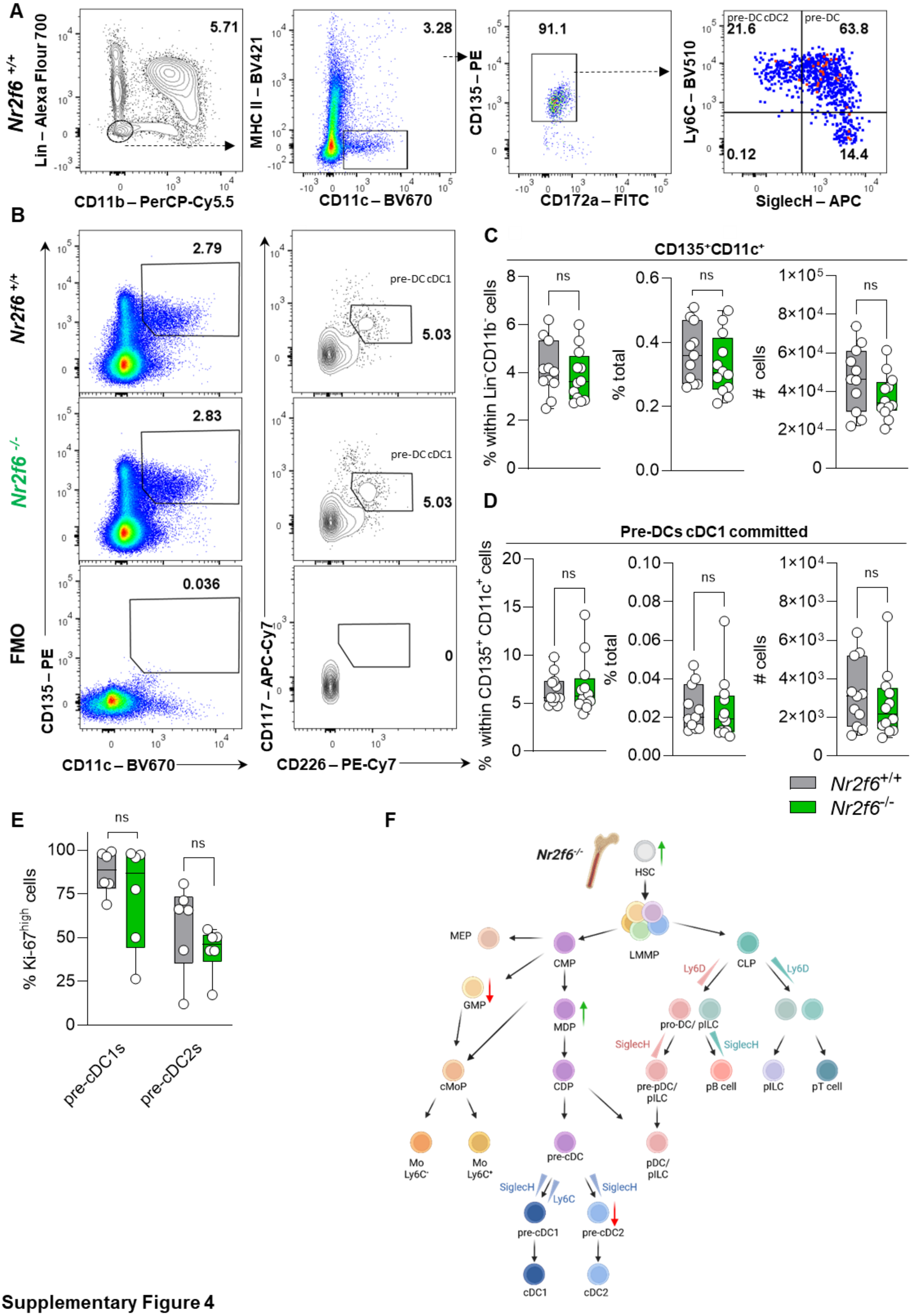

Supplementary Figure 4

Supplementary Figure 4: Pre-cDC progenitor populations are not altered in *Nr2f6*-deficient mice.

(A) Representative dot plots of the gating scheme of the bone marrow-derived pre-DC and pre-cDC cell populations from wild-type ($Nr2f6^{+/+}$) mice.

(B) Representative dot plots of the gating scheme of the bone marrow-derived pre-DC cDC1 cell populations from wild-type ($Nr2f6^{+/+}$) or *Nr2f6*-deficient ($Nr2f6^{-/-}$) mice and FMO controls for CD135 and CD226.

(C-D) Quantification of the percent of parent, percent of total and total cell numbers of live $(Lin)^{-}CD11b^{int}$ $CD11c^{+}CD135^{+}$ **(C)** and $CD226^{+}CD117^{int}$ pre DCs (pre-cDC1 committed) **(D)** populations of wild-type ($Nr2f6^{+/+}$) or *Nr2f6*-deficient ($Nr2f6^{-/-}$) mice.

(E) Quantification of the percent Ki-67^{+} pre-cDC1 and pre-cDC2 commited cell populations from bone marrow of wild-type ($Nr2f6^{+/+}$) or *Nr2f6*-deficient ($Nr2f6^{-/-}$) mice.

(F) Schematic representation of hematopoietic stem cells (HSCs), myeloid, and lymphoid progenitors in the bone marrow of *Nr2f6*-deficient mice. Scheme modified after Dress ([27]). Created with BioRender.com.

Representative data are shown as pooled experiments of at least two independent experiments, total n = 6-11/6-11 ($Nr2f6^{+/+}$)/($Nr2f6^{-/-}$). Each dot represents the data of an individual mouse. Results are shown median ± IQR with whiskers from min. to max. The Shapiro-Wilk test evaluated the normality of data. Asterisks indicate statistically significant differences between genotypes calculated using the Mann-Whitney *U*, Student's or Welch's corrected t-test. A p-value < 0.05 was considered statistically significant.

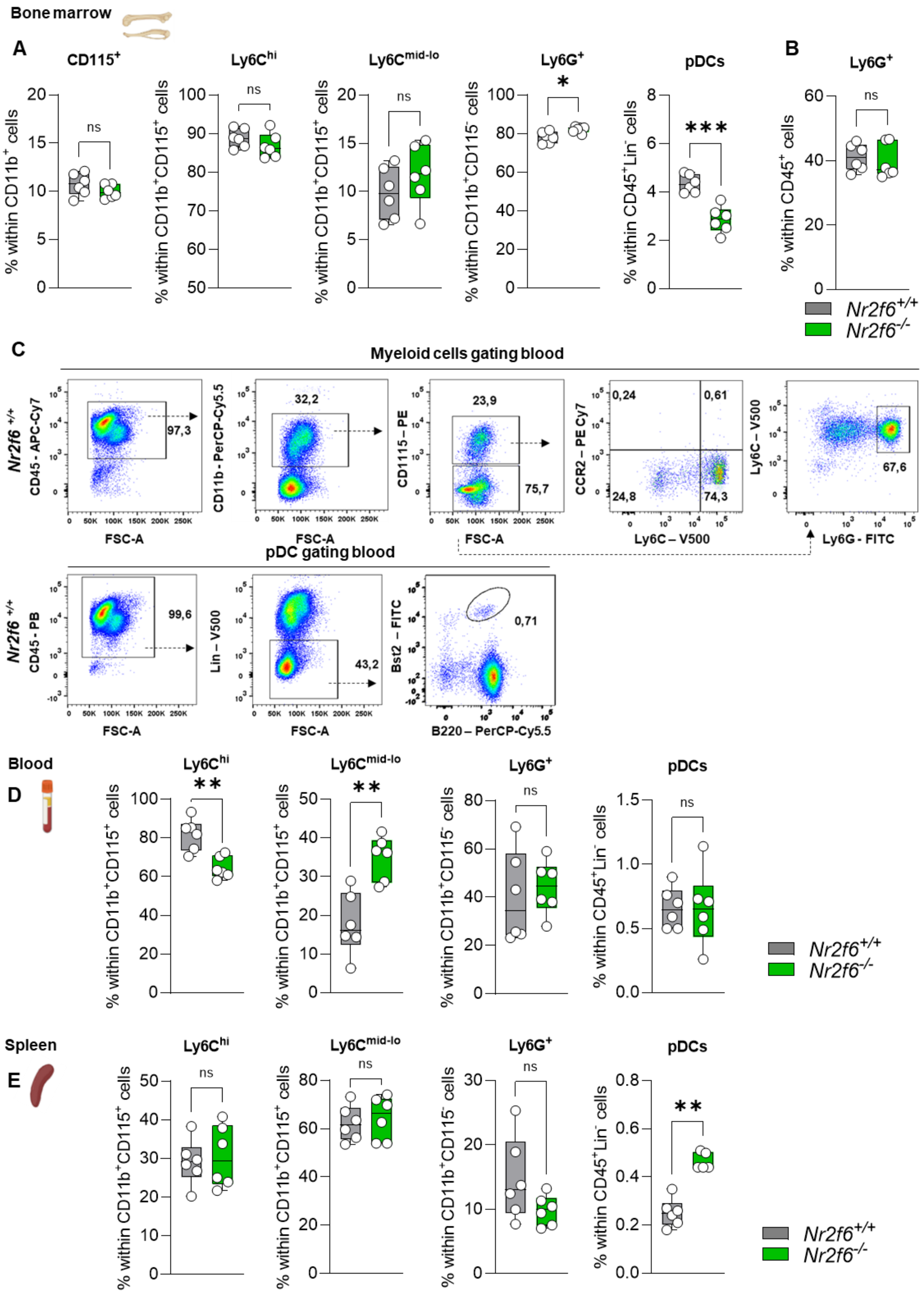

Supplementary Figure 5

Supplementary Figure 5:

(A) Quantification of percent of parent monocytes ($CD115^+$), classical $Ly6C^{hi}$ monocytes, non-classical $Ly6C^{mid\text{-}lo}$ monocytes, $Ly6G^+$ neutrophils and $Bst2^+B220^+$ pDCs in the bone marrow from wild-type wild-type (*Nr2f6*$^{+/+}$) or *Nr2f6*-deficient (*Nr2f6*$^{-/-}$) mice.

(B) Quantification of percent of total $Ly6G^+$ neutrophils in the bone marrow from wild-type (*Nr2f6*$^{+/+}$) or *Nr2f6*-deficient (*Nr2f6*$^{-/-}$) mice.

(C-D) Gating scheme **(C)** and quantification **(D)** of the percent of parent of classical $Ly6C^{hi}$ monocytes, non-classical $Ly6C^{mid\text{-}lo}$ monocytes, $Ly6G^+$ neutrophils and $Bst2^+B220^+$ pDCs in the blood of wild-type (*Nr2f6*$^{+/+}$*)* mice or *Nr2f6*-deficient (*Nr2f6*$^{-/-}$) mice.

(E) Quantification of percent of parent classical $Ly6C^{hi}$ monocytes, non-classical $Ly6C^{mid\text{-}lo}$ monocytes, $Ly6G^+$ neutrophils and $Bst2^+B220^+$ pDCs in the spleen from wild-type (*Nr2f6*$^{+/+}$) or *Nr2f6*-deficient (*Nr2f6*$^{-/-}$) mice.

Representative data are shown as pooled experiments of two independent experiments, total n = 5-6/6 (*Nr2f6*$^{+/+}$)/(*Nr2f6*$^{-/-}$). Each dot represents the data of an individual mouse. Results are shown as median ± IQR with whiskers from min. to max. The Shapiro-Wilk test evaluated the normality of data. Outliers were excluded using ROUT (Q=1%) in GraphPad Prism. Asterisks indicate statistically significant differences between genotypes calculated using the Mann-Whitney *U*, or the Student's *t*-test, or . A *p*-value < 0.05 was considered statistically significant*0.05, **0.01, ***0.001.

Supplementary Table 1: Markers used to identify stem and progenitor populations in the bone marrow

Population	Lineage	Pre-gate	Key markers
LT-HSC	(CD3 CD19 Ly6G Ter-119 NK1.1 CD127)$^-$	Sca-1$^+$c-Kit$^+$	CD135$^-$CD48$^-$CD150$^+$
ST-HSC	(CD3 CD19 Ly6G Ter-119 NK1.1 CD127)$^-$	Sca-1$^+$c-Kit$^+$	CD135$^-$CD48$^-$CD150$^-$
HSPC	(CD3 CD19 Ly6G Ter-119 NK1.1 CD127)$^-$	Sca-1$^+$c-Kit$^+$	CD135$^-$
LMPP	(CD3 CD19 Ly6G Ter-119 NK1.1 CD127)$^-$	Sca-1$^+$c-Kit$^+$	CD135$^+$CD150$^-$
MPP2	(CD3 CD19 Ly6G Ter-119 NK1.1 CD127)$^-$	Sca-1$^+$c-Kit$^+$	CD135$^-$CD48$^+$CD150$^+$
MPP3	(CD3 CD19 Ly6G Ter-119 NK1.1 CD127)$^-$	Sca-1$^+$c-Kit$^+$	CD135$^-$CD48$^+$CD150$^-$
MPP4	(CD3 CD19 Ly6G Ter-119 NK1.1 CD127)$^-$	Sca-1$^+$c-Kit$^+$	CD135$^+$CD48$^+$CD150$^-$
LSK	(CD3 CD19 Ly6G Ter-119 NK1.1 CD127)$^-$	Sca-1$^+$c-Kit$^+$	
GMP	(CD3 CD19 Ly6G Ter-119 NK1.1 CD127)$^-$	Sca-1$^-$c-Kit$^+$CD115$^-$	CD16/32$^+$
CMP	(CD3 CD19 Ly6G Ter-119 NK1.1 CD127)$^-$	Sca-1$^-$c-Kit$^+$CD115$^-$	CD16/32$^-$CD34$^+$
MEP	(CD3 CD19 Ly6G Ter-119 NK1.1 CD127)$^-$	Sca-1$^-$c-Kit$^+$CD115$^-$	CD16/32$^-$CD34$^-$
CLP	(CD3 CD19 Ly6G Ter-119 NK1.1)$^-$	Sca-1$^+$c-Kit^{lo-int}	CD135$^+$CD127$^+$
pre pro Nka	(CD3 CD19 Ly6G Ter-119 NK1.1)$^-$	Sca-1$^+$c-Kit^{lo-int}	CD135$^+$CD127$^-$
MDP	(CD3 CD19 Ly6G Ter-119 NK1.1 B220 CD11b)$^-$	CD115$^+$	CD117$^+$CD135$^+$
CDP	(CD3 CD19 Ly6G Ter-119 NK1.1 B220 CD11b)$^-$	CD115$^+$	CD117^{lo-int}CD135$^+$
cMoP	(CD3 CD19 Ly6G Ter-119 NK1.1 B220 CD11b)$^-$	CD115$^+$CD117$^+$CD135$^-$	Ly6C$^+$
pre-DC cDC1-commited	(CD3 CD19 Ly6G Ter-119 NK1.1 B220 CD11b)$^-$	CD135$^+$CD11c$^+$	CD117^{int}/CD226$^+$
pre-DC cDC2-commited	(CD3 CD19 Ly6G Ter-119 NK1.1 B220 CD11b)$^-$	CD11c$^+$MHCII$^-$ CD135$^+$CD172a$^-$	Ly6C$^+$SiglecH$^-$
pre-DC	(CD3 CD19 Ly6G Ter-119 NK1.1 B220 CD11b)$^-$	CD11c$^+$MHCII$^-$ CD135$^+$CD172a$^-$	Ly6C$^{+/-}$SiglecH$^+$

Supplementary Table 2: Antibodies used for flow cytometry

Antigen	Fluorochrome	Clone	Manufacturer	Order number	Dilution
B220	Biotin	RA3-6B2	BioLegend	103204	1:200
B220	PerCP-Cy5.5	RA3-6B2	BioLegend	103236	1:200
Bst2	FITC	927	BioLegend	127007	1:200
CCR2	PeCy7	QA18A56	BioLegend	160107	1:200
CD115	BV711	AFS98	BioLegend	135515	1:100
CD115	PE	AFS98	BioLegend	135505	1:200
CD11b	PerCP5.5	M1/70	BioLegend	101227	1:400
CD11c	BV510	N418	BioLegend	117339	1:400
CD127	PeCy7	A7R34	BioLegend	135013	1:100
CD135	APC	A2F10	BioLegend	135309	1:100
CD135	PE	A2F10	BioLegend	135306	1:100
CD150	BV786	TC15-12F12.2	BioLegend	115941	1:200
CD16/32	BV421	93	BioLegend	101332	1:200
CD172a	FITC	P84	BioLegend	144005	1:200
CD19	Biotin	6D5	BioLegend	115503	1:200
CD226	PeCy7	10E5	BioLegend	128811	1:100
CD3	Biotin	145-2C11	BioLegend	100304	1:200
CD34	FITC	REA383	Miltenyi Biotec	130-117-775	1:100
CD45	Pacific Blue	30-F11	BioLegend	103126	1:200
CD48	PerCP5.5	HM48-1	BioLegend	103421	1:400
c-Kit	APC-Cy7	2B8	BioLegend	105826	1:200
Ly6C	BV510	HK1.4	BioLegend	128033	1:200
Ly6G	Biotin	RB6-8C5	BioLegend	108404	1:200
Ly6G	FITC	1A8	BioLegend	127625	1:200
MHC-II	BV421	M5/114.15.2	BioLegend	107632	1:100
NK1.1	Biotin	PK136	BioLegend	108704	1:100
Sca-1	PE	D7	BioLegend	108107	1:200
SiglecH	APC	eBio440c	eBioscience	17-0333-82	1:400
Ter-119	Biotin	TER-119	BioLegend	116204	1:200
Ki-67	Pe-Dazzle	16A8	BioLegend	652427	1:100
